# Multi-Omics Deciphers Divergent Mechanisms in Differentially Cardiac-Remodeled Yili Horses Under Conditions of Equivalent Power Output

**DOI:** 10.3390/ani15223251

**Published:** 2025-11-09

**Authors:** Tongliang Wang, Xixi Yang, Wanlu Ren, Jun Meng, Xinkui Yao, Hongzhong Chu, Runchen Yao, Manjun Zhai, Yaqi Zeng

**Affiliations:** 1 College of Animal Science, Xinjiang Agricultural University, Urumqi 830052, China; wtl13639911402@163.com (T.W.); xxyang2022@126.com (X.Y.); renwanlu@xjau.edu.cn (W.R.); junm86@xjau.edu.cn (J.M.); yaoxinkui@xjau.edu.cn (X.Y.); zhaimanjun@yeah.net (M.Z.); 2 Xinjiang Key Laboratory of Horse Breeding and Exercise Physiology, Urumqi 830052, China; 3 Horse Industry Research Institute, Xinjiang Agricultural University, Urumqi 830052, China; 4 Xinjiang Yili Kazakh Autonomous Prefecture Animal Husbandry Station, Urumqi 835000, China; 13364712998@163.com (H.C.); m18095936088@163.com (R.Y.)

**Keywords:** Yili horse, cardiac remodeling, integrated analysis, same racing load

## Abstract

**Simple Summary:**

This study aimed to investigate the molecular response mechanisms of Yili horses with different degrees of cardiac remodeling under the same exercise intensity. Twenty 2-year-old Yili horses were divided into a high cardiac remodeling group (BH, with parameters such as EDV > 500 mL) and a low cardiac remodeling group (BL, with parameters such as EDV < 450 mL) based on echocardiographic parameters. Blood samples were collected before and after a 1000 m constant-speed exercise, followed by metabolomic, transcriptomic, and miRNA analyses. The results showed that the BH group exhibited stronger post-exercise lipid mobilization and significant enrichment of the sphingolipid signaling pathway; core miRNAs (e.g., let-7 family, miR-186) and their target genes (e.g., ALAS2) regulated cardiac electrophysiology and energy metabolism. Integrated multi-omics analysis revealed that the BH group-maintained energy homeostasis through the glycine–serine–threonine metabolic pathway and phosphatidylserines (e.g., PS (17:0_16:1)), providing a basis for understanding the differences in equine cardiac adaptation to exercise.

**Abstract:**

Exercise performance is a critical trait for evaluating the economic and breeding value of working and athletic horses, with cardiac structure and function serving as essential physiological determinants of athletic capacity. This study aimed to investigate the multi-omics response mechanisms associated with varying degrees of cardiac remodeling under identical exercise intensity. Twenty 2-year-old Yili horses were selected and categorized based on echocardiographic parameters into a high cardiac remodeling group (BH; EDV > 500 mL, SV > 350 mL, EF > 66%) and a low cardiac remodeling group (BL; EDV < 450 mL, SV < 330 mL, EF < 64%). Blood samples were collected before and after the 1000 m constant-speed test (pre-test high cardiac remodeling group (BH, *n* = 10), post-test high cardiac remodeling group (AH, *n* = 10), pre-test low cardiac remodeling group (BL, *n* = 10), post-test low cardiac remodeling group (AL, *n* = 10)), and integrated metabolomic, transcriptomic, and miRNA profiling were conducted to systematically characterize molecular responses to exercise-induced stress. Metabolomic analysis identified a total of 1936 lipid metabolites, with the BH group exhibiting stronger post-exercise lipid mobilization and significant enrichment of sphingolipid signaling pathways. Transcriptomic and miRNA analyses further revealed that key miRNAs in the BH group, including miR-186, miR-23a/b, and the let-7 family, along with their target genes (e.g., GNB4, RGS5, ALAS2), were involved in fine regulation of cardiac electrophysiology, oxidative stress, and energy metabolism. Integrated analysis indicated that the AH vs. BH comparison uniquely enriched pathways related to glycine-serine-threonine metabolism and glycosylphosphatidylinositol (GPI)-anchor biosynthesis, whereas the AL vs. BL comparison showed unique enrichment of α-linolenic acid and arachidonic acid metabolism pathways. Ultimately, multi-omics integration identified that in the BH group, eca-let-7d, eca-let-7e, eca-miR-196b, eca-miR-2483, and eca-miR-98 regulate ALAS2 and, together with GCSH, influence the enrichment of lipids such as PS(17:0_16:1), PS(18:0_18:1), and PS(20:0_18:1). These lipids participate in glycine, serine, and threonine metabolism through complex pathways, collectively modulating energy supply, inflammatory responses, and muscle function during exercise. This study reveals the molecular mechanisms by which horses with high cardiac remodeling maintain energy homeostasis and myocardial protection during exercise.

## 1. Introduction

The athletic performance is one of the key selection criteria for breeding sport horses. The heart, as the central driver of the circulatory system, serves as a critical physiological determinant of athletic capacity. During long-term domestication and selective breeding, horses exhibit pronounced cardiac adaptive remodeling; frequently trained individuals often develop a “large heart” phenotype [1], which correlates positively with superior endurance and recovery capacity. However, in practice, significant variations in the degree of cardiac remodeling are observed even among horses subjected to identical training regimens. Whether these differences reflect fundamentally distinct physiological and molecular responses to equivalent exercise intensity remains a crucial scientific question in the field of equine exercise physiology.

Current research on equine exercise primarily focuses on two core competition types: endurance races and flat speed races [2]. Previous studies have demonstrated that horses with differing competitive performance exhibit significant variations in the composition and concentration of blood, urine, and saliva metabolites following chronic or acute exercise [3,4]. These investigations have further identified potential omics-level biomarkers before and after exercise, providing key theoretical support and practical references for the scientific prediction of elite racing performance [5,6]. Nevertheless, existing studies generally overlook the regulatory mechanisms underlying how differences in cardiac remodeling modulate systemic material mobilization under the same exercise intensity.

In recent years, integrated multi-omics approaches have provided novel insights into the molecular mechanisms of cardiac exercise adaptation. MicroRNAs (miRNAs), as critical post-transcriptional regulators, play essential roles in exercise-induced cardiac remodeling. Studies have shown that exercise dynamically modulates the expression of multiple cardiac miRNAs, such as miR-1, miR-133, miR-21, miR-144, and miR-145, which participate in cardiac hypertrophy through the regulation of signaling pathways including PI3K/Akt/mTOR [7,8]. Concurrently, changes in messenger RNA (mRNA) expression profiles reflect gene regulatory networks in the heart during exercise, encompassing biological processes such as energy metabolism, cell proliferation, and stress response [9]. Metabolomic analyses further reveal that post-exercise levels of cardiac metabolites including branched-chain amino acids, 3-hydroxybutyrate, and sphingolipids undergo significant alterations, with some changes exhibiting sex-dependent differences, indicating that metabolic reprogramming constitutes a fundamental basis for cardiac adaptation to exercise [10,11].

Although progress has been made in previous studies, most research has focused on a single omics layer or relatively homogeneous populations, failing to reveal the multi-omics response characteristics of individuals with different heart sizes under equivalent exercise intensity. Variations in cardiac size may correspond to differences in energy demand, stress thresholds, and molecular regulatory patterns, yet the underlying mechanisms remain unclear, limiting our understanding of individual-specific cardiac adaptation to exercise. Therefore, this study aims to integrate miRNA, transcriptomic, and metabolomic analyses to investigate the multi-omics response differences in horses with varying degrees of cardiac remodeling under identical exercise intensity.

## 2. Materials and Methods

### 2.1. Ethical Statement

This study was approved by the Animal Policy and Welfare Committee of Xinjiang Agricultural University (Ethical Approval No.: 2023037), and all experimental procedures were conducted in strict accordance with relevant animal welfare and ethical regulations. Informed consent was obtained from all horse owners.

### 2.2. Experimental Design and Horse Grouping

Twenty 2-year-old Yili horses were selected from the Yili Local State-owned Stud Farm, Xinjiang Uygur Autonomous Region. To minimize confounding factors, the horses were matched for birth date (ensuring consistent growth stage), exhibited no significant differences in body conformation, and were maintained under a standardized feeding and management regimen (including diet, feeding schedule, and exercise management) throughout the study to ensure uniform baseline physiological status.

Repeated assessments of cardiac structure and function were conducted, including key parameters such as left ventricular end-diastolic diameter (LVID), end-diastolic volume (EDV), stroke volume (SV), and ejection fraction (EF). Based on characteristic features of physiological cardiac remodeling such as the extent of ventricular eccentric dilation, adaptive wall thickening, and enhanced pumping efficiency horses were categorized into two groups: a high cardiac remodeling group with LVIDd > 9.5 cm, LVIDs > 5.6 cm, EDV ≥ 500 mL, SV ≥ 350 mL, and EF ≥ 66%, and a low cardiac remodeling group with LVIDd < 9.2 cm, LVIDs < 5.5 cm EDV ≤ 450 mL, SV ≤ 330 mL, and EF ≤ 64% (The grouping criteria in this study specifically refer to the previous research findings of our team [12]). Both groups were matched for age, body weight, body measurements, and baseline training level, ensuring comparable exercise load during the subsequent 1000 m race and statistical validity for inter-group comparisons. The horses were further assigned into pre-race high remodeling (BH, *n* = 10), post-exercise high remodeling (AH, *n* = 10), pre-race low remodeling (BL, *n* = 10), and post-exercise low remodeling (AL, *n* = 10) groups, maintaining equal sample sizes for robust statistical analysis.

Subsequently, all horses completed a 1000 m speed race under the guidance of experienced jockeys. The race intensity was controlled to reach a heart rate of 180–200 bpm for each horse (The heart rate of the horses and exercise intensity were monitored using a heart rate sensor with heart rate straps (Polar H10, Polar Electro Oy, Kempele, Finland)), representing a vigorous exercise range [13]. The speed is approximately 12.5 m per second.

### 2.3. Echocardiographic Detection of Cardiac Structure and Function

A Mindray M6 color Doppler ultrasound system (Shenzhen, China) was employed to acquire and analyze echocardiograms of horses at rest for quantitative assessment of cardiac structure and function. The detailed measurement method can be found in Supplementary Text S1.

### 2.4. Blood Sample Collection

All horses in this study underwent a unified fasting protocol (12 h fasting with free access to water) before pre- and post-exercise blood collection. After disinfecting the sampling site with alcohol, blood was collected via jugular vein puncture. Samples were immediately transferred into K2-EDTA tubes for hematological analysis. From fasted horses, 15 mL of jugular vein blood was collected: 5 mL was combined with TRIzol reagent at a 1:3 ratio for RNA extraction, and another 5 mL was reserved for metabolomic analysis.

### 2.5. Metabolomic Analysis: Experimental Design and Methods

#### 2.5.1. Plasma Lipidomic Detection (UPLC-MS/MS)

Plasma lipids were analyzed both qualitatively and quantitatively using ultra-performance liquid chromatography-tandem mass spectrometry (UPLC-MS/MS). Following protein precipitation and lipid extraction, the resulting supernatants were subjected to analysis. Chromatographic separation was carried out on an ExionLC™ AD UPLC system (SCIEX, Framingham, MA, USA) equipped with a C30 column (2.6 μm, 2.1 mm × 100 mm) (Thermo Fisher Scientific, Waltham, MA, USA). The mobile phases were A, acetonitrile/water (60:40, *v*/*v*) containing 0.1% formic acid and 10 mM ammonium formate; and B, acetonitrile/isopropanol (10:90, *v*/*v*) containing 0.1% formic acid and 10 mM ammonium formate. Gradient elution was applied as follows: 0 min (80:20 A/B) → 2.0 min (70:30) → 4.0 min (40:60) → 9.0 min (15:85) → 14.0 min (10:90) → 15.5 min (5:95) → 17.3 min (5:95) → 17.3–20.0 min (80:20, re-equilibration). The flow rate was set at 0.35 mL/min, the column temperature at 45 °C, and the injection volume was 2 μL.

Lipid identification and quantification were performed on a QTRAP^®^ 6500+ triple quadrupole-linear ion trap mass spectrometer (SCIEX, USA) with an ESI Turbo ion spray interface in both positive and negative ion modes, controlled by Analyst 1.6.3 software (SCIEX, Framingham, MA, USA). Lipid concentrations were determined using the AB Sciex QTRAP 6500 LC-MS/MS platform in combination with the MetWare detection system (http://www.metware.cn/; 10 September 2025), and qualitative validation was conducted against reference standards.

#### 2.5.2. Data Preprocessing

Raw mass spectrometry data were processed using Analyst 1.6.3 (SCIEX) for peak extraction, integration, and baseline correction. Relative concentrations of metabolites were calculated based on the ratios of their integrated peak areas. Quality control (QC) samples, prepared as equal-volume mixtures of all study samples, were used to assess instrument stability and data reliability. Pearson correlation analysis and coefficient of variation (CV) statistics for QC samples indicated that over 85% of compounds had CVs < 0.5, and more than 75% had CVs < 0.3, demonstrating stable and reproducible measurements.

#### 2.5.3. PCA and OPLS-DA for Differential Lipid Screening

Unsupervised principal component analysis (PCA) was performed on the preprocessed data using the prcomp function in R 4.3.3 (www.r-project.org; 10 September 2025) to evaluate inter-group lipid metabolic differences and intra-group variation.

Orthogonal partial least squares discriminant analysis (OPLS-DA) was conducted using the MetaboAnalystR package and OPLSR after centering and scaling the data. Variable importance in projection (VIP) scores were derived from the OPLS-DA model. Differential lipids were identified based on the criteria: VIP > 1, *p* < 0.05, and fold change (FC) ≥ 2 or ≤0.5.

#### 2.5.4. KEGG Enrichment Analysis of Differentially Expressed Metabolites

Lipids were annotated against the KEGG database, and differentially expressed metabolites (DEMs) were further characterized. Volcano plots were generated using the ggplot2 package, and z-score normalized hierarchical clustering heatmaps were constructed with the pheatmap package to visualize DEM expression patterns. KEGG enrichment bubble plots (ggplot2) were constructed to analyze DEM-related pathways. Enrichment was defined as (x/n) > (y/n~) (x: DEMs in pathway; n: total annotated metabolites in pathway; y: all detected lipids in pathway), and significant enrichment at *p* < 0.05.

### 2.6. Transcriptomics Analysis

#### 2.6.1. RNA Extraction and Library Construction

RNA concentration was measured using a Qubit 4.0 fluorometer v2.19(Thermo Fisher Scientific, Waltham, MA, USA), RNA integrity was assessed with a Qsep400 bioanalyzer (BiOptic Inc., New Taipei City, Taiwan, China) (Supplementary Table S1). High-quality RNA was used for library preparation. mRNA molecules with poly(A) tails were enriched using Oligo(dT) magnetic beads and then fragmented with a fragmentation buffer. First-strand cDNA was synthesized using random hexamers and the fragmented RNA as a template, followed by second-strand cDNA synthesis with buffer, dNTPs, and DNA polymerase. The resulting double-stranded cDNA was purified using DNA purification beads, subjected to end repair, A-tailing, and adapter ligation. Fragment size selection was performed with DNA purification beads, and final cDNA libraries were obtained through PCR enrichment.

#### 2.6.2. Library Quality Control and Sequencing

Preliminary library quantification was performed using the Qubit dye method, and insert sizes were assessed with a fragment analyzer. Libraries that met the expected insert size criteria were further quantified by qPCR, with effective concentrations > 2 nM. Libraries were pooled according to the target sequencing depth and sequenced on the Illumina platform (Supplementary Text S2).

#### 2.6.3. Bioinformatics Analysis

Filtered clean reads were aligned to the reference genome (GCF_002863925.1_EquCab3.0_genomic.fna.gz) for structural analysis, including alternative splicing detection, novel gene discovery, and gene structure optimization, as well as for expression analysis, including differential expression, functional annotation, and functional enrichment.

#### 2.6.4. Differential Gene Screening

For samples with biological replicates, DESeq2 1.42.0was used for inter-group differential expression analysis. *p*-values were adjusted using the Benjamini–Hochberg method to control the false discovery rate (FDR). Differentially expressed genes were identified using the criteria |log_2_Fold Change| ≥ 1 and *p* < 0.05 (Supplementary Text S3).

#### 2.6.5. Functional Annotation and Pathway Analysis of DE mRNAs

Gene Ontology (GO) enrichment analysis of differentially expressed genes (DEGs) was performed using GOseq (release 2.12), while KEGG pathway enrichment analysis was conducted with KOBAS (v2.0) using the KEGG database. Enrichment was considered significant at *p* < 0.05. Corresponding visualizations, including charts and heatmaps, were generated to illustrate the results.

#### 2.6.6. Isolation, Library Construction, Sequencing, and Identification of miRNAs

Total RNA was extracted from blood using a procedure similar to that for mRNA, except that size-exclusion chromatography was applied to isolate small RNAs. Specific adapters were ligated to the 3′ and 5′ ends of the miRNAs, followed by reverse transcription into cDNA and amplification with adapter-specific primers. After removal of 3′ adapters and junk sequences, small cDNAs of 18–26 nucleotides were filtered and aligned to the mRNA, RFam, and Repbase databases. Reads passing length and database filters were used for miRNA identification with ACGT101-miR (v4.2). Potential miRNAs were annotated using miRBase and the reference genome (Supplementary Text S4. Differentially expressed miRNAs (DEmiRNAs) were identified at *p* < 0.05) (Supplementary Text S5).

#### 2.6.7. Target Gene Prediction of DEmiRNAs

Target genes of DEmiRNAs were predicted using both TargetScan (v5.0) and miRanda (v3.3a) for cross-validation. For TargetScan, genes with a context score percentile < 50 were excluded (retaining those ≥50). For miRanda, genes with maximum energy > −10 kcal/mol were excluded (retaining those <−10 kcal/mol). The intersection of candidate target genes identified by both tools (TargetScan score ≥ 50 and miRanda energy < −10 kcal/mol) was used as the final set of DEmiRNA target genes.

### 2.7. Integration of Transcriptomic and Metabolomic Data

Principal component analysis (PCA) was performed separately on the transcriptomic (DEGs) and metabolomic (DEMs) datasets to assess data reliability. KEGG pathway enrichment analyses were conducted for both DEGs and DEMs, and overlapping pathways were identified to elucidate potential “gene–metabolite” co-regulation mechanisms.

### 2.8. miRNA Isolation, cDNA Library Construction, and Sequencing Identification

All figures were generated using GraphPad Prism 8.0 (GraphPad Software, San Diego, CA, USA). Statistical analyses were performed with SPSS 26.0 (IBM, Armonk, NY, USA), and data are presented as mean ± standard deviation (Mean ± SD). Inter-group differences were evaluated using one-way ANOVA, with a test for homogeneity of variance. When variances were unequal, Welch’s correction was applied. A significance threshold of *p* < 0.05 was used.

### 2.9. RT-qPCR Validation of mRNA and miRNA

The transcriptomic data utilized in the present study originated from a 2023 research project. This project focused on two main aspects: first, exploring how long-term exercise impacts the cardiac structure and function of horses; second, conducting profiling analyses of blood transcriptomes and plasma lipidomes. Detailed information regarding sample preparation procedures, experimental treatment protocols, and study background has been previously published in relevant research articles.

All samples included in this study were processed in accordance with standard procedures after sequencing. Additionally, initial validation via quantitative polymerase chain reaction (qPCR) was performed on these samples. It should be noted that no residual sample material was left for further validation experiments.

The raw sequencing data generated in this study have been deposited in the Sequence Read Archive (SRA) database of the National Center for Biotechnology Information (NCBI), with the accession number SUB15457665. For comprehensive protocols covering sample preparation, sequencing operations, and qPCR verification processes, refer to the dissertation [14].

### 2.10. Statistical Analysis

All charts were created with GraphPad Prism 8.0 (GraphPad Software Inc., San Diego, CA, USA). Data statistical analyses were conducted via SPSS 26.0 (IBM, Armonk, NY, USA). Data were presented as mean ± standard deviation. Inter-group differences were assessed by one-way ANOVA. Tests for within-group variance homogeneity were performed, where *p* > 0.05 suggested no significant discrepancy.

## 3. Results

### 3.1. Echocardiographic Parameters of Horses

**Figure 1:**
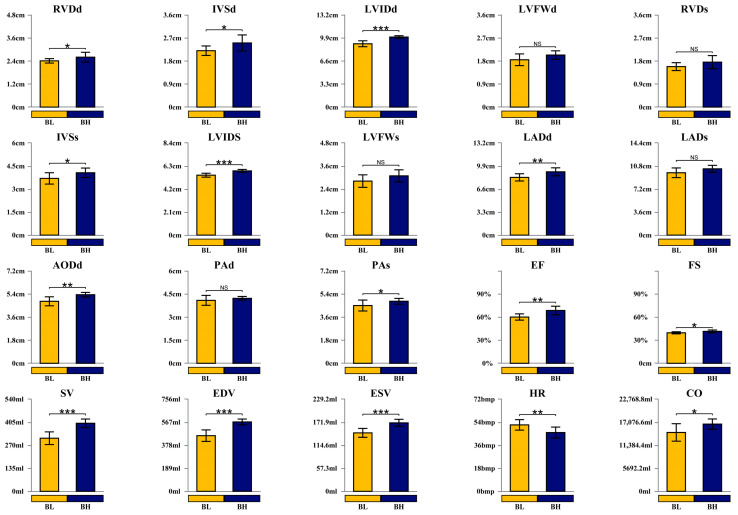
Bar chart of differences in cardiac structure and function parameters: before-race high physiological remodeling group, BH (*n* = 10); before-race low physiological remodeling group, BL (*n* = 10). RVDd: End-diastolic right ventricular diameter; IVSd: End-diastolic interventricular septal thickness; LVIDd: End-diastolic left ventricular diameter; LVFWd: End-diastolic left ventricular free wall thickness; LADd: End-diastolic left atrial diameter; LADs: End-systolic left atrial diameter; AODd: End-diastolic aortic root diameter; PAd: End-diastolic pulmonary artery diameter; PAs: End-systolic pulmonary artery diameter; EF: Ejection fraction; FS: Fractional shortening; SV: Stroke volume; EDV: End-diastolic left ventricular volume; ESV: End-systolic left ventricular volume; CO: Cardiac output; HR: Heart rate. Asterisks indicate statistically significant differences between BH and BL under identical growth conditions (NS *p* > 0.05, * *p* < 0.05, ** *p* < 0.01, *** *p* < 0.001).

The cardiac structure and function of the BL and BH groups are presented in Figure 1. Compared with the BL group, horses in the BH group exhibited significantly greater LVIDd, LVIDs, SV, EDV, and ESV (*p* < 0.001), as well as larger LADd, AODd, and EF (*p* < 0.01). RVDd, IVSd, IVSs, PAs, FS, and CO were also significantly higher in the BH group (*p* < 0.05). No significant differences were observed between the two groups for LVFWd, RVDs, LVFWs, LADs, or PAd (*p* > 0.05).

### 3.2. Metabolomic Analysis

**Figure 2:**
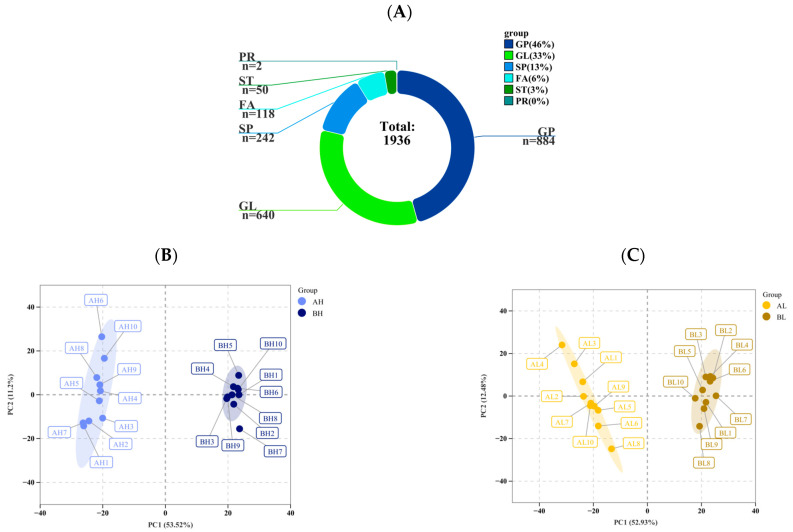

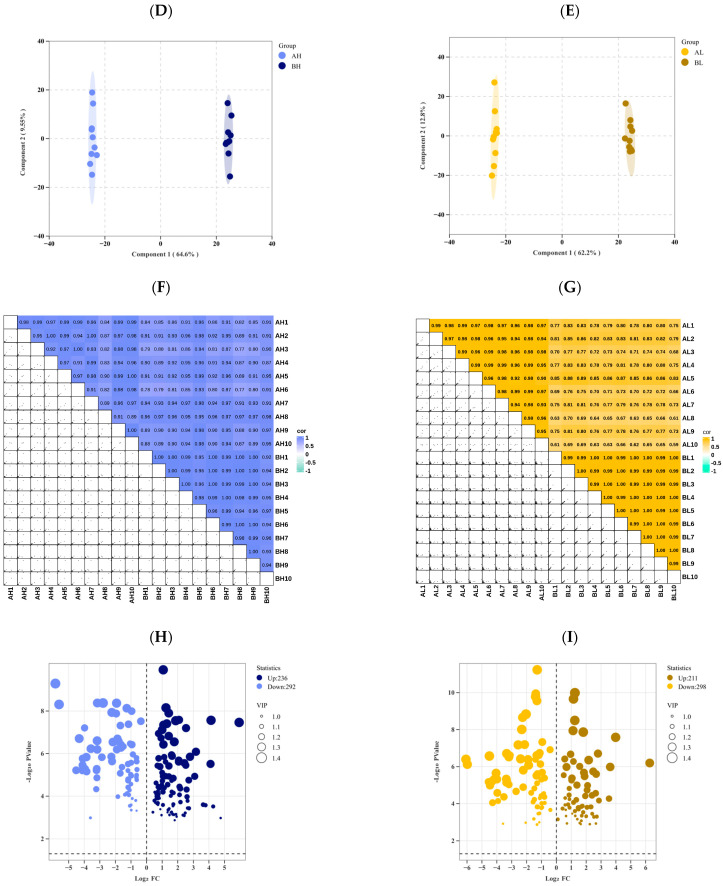

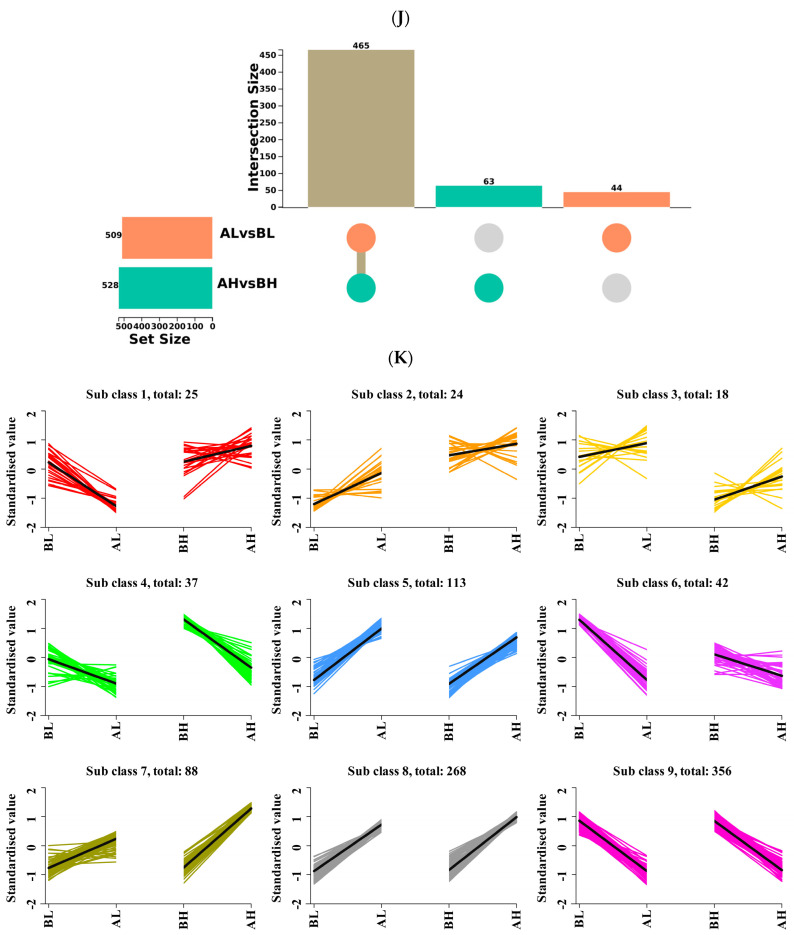

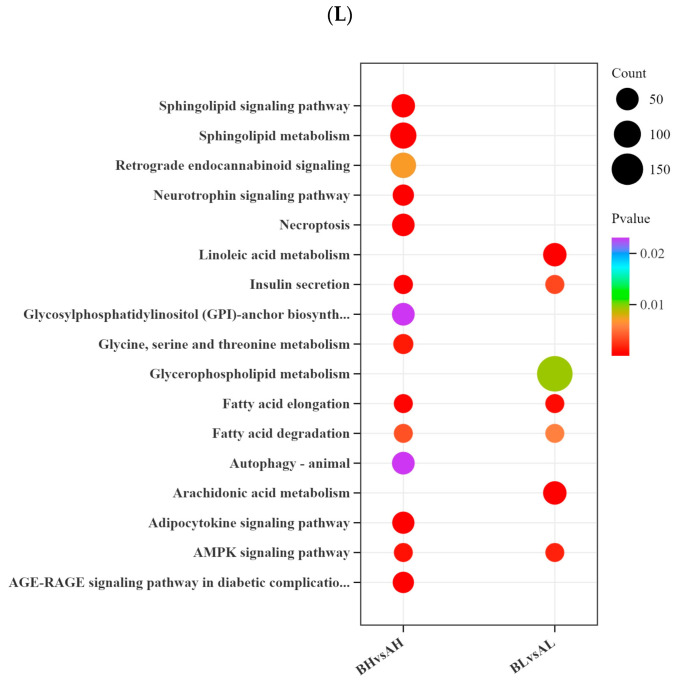
(**A**) Summary of lipid classes. The pie chart displays the total number of metabolites detected and their classification. (Note: Due to rounding of individual percentage values to the nearest integer, the total sum is 101%. The original unrounded data sum to 100%.) (**B**) PCA score plot of AH vs. BH. (**C**) PCA score plot of AL vs. BL. In the PCA plots, PC1 and PC2 represent the first and second principal components, respectively, and the percentage indicates the proportion of variance explained by each component. (**D**) OPLS-DA score plot of AH vs. BH. (**E**) OPLS-DA score plot of AL vs. BL. In the OPLS-DA plots, the *x*-axis represents the predictive component, while the *y*-axis represents the orthogonal component; the percentage indicates the explanatory power of each component for the dataset. (**F**) Correlation matrix showing pairwise relationships among samples/metabolites in AH vs. BH. (**G**) Correlation matrix showing pairwise relationships among samples/metabolites in AL vs. BL. The upper right panels display correlation heatmaps of different variables, while the lower left panels show scatter plots illustrating pairwise correlations. (**H**) Volcano plot of AH vs. BH. (**I**) Volcano plot of AL vs. BL. (**J**) Advanced Venn diagram of differential metabolites before and after exercise in different groups, illustrating shared and unique metabolites under exercise-induced stress. (**K**) k-means clustering analysis of differential lipids before and after exercise in different groups. The *x*-axis represents pre- and post-exercise states, and the *y*-axis shows the standardized Z-score of each lipid. Colored lines indicate the dynamic expression of individual metabolites, while the black line represents the average expression trend of each cluster. (**L**) KEGG pathway enrichment bubble plot of differential lipids before and after exercise in different groups. The *x*-axis denotes the comparison groups, and the *y*-axis indicates the enriched metabolic pathways. Note that due to layout requirements, the two incompletely displayed pathways in (**L**) are AGE-RAGE signaling pathway in diabetic complications and Glycosylphosphatidylinositol (GPI)-anchor biosynthesis.

To investigate metabolic alterations associated with different degrees of cardiac remodeling under exercise-induced stress, plasma metabolomic profiling was conducted using ultra-high-performance liquid chromatography–mass spectrometry (UHPLC–MS/MS). A total of 1936 lipid metabolites were identified, including 884 glycerophospholipids (GP), 640 glycerolipids (GL), 242 sphingolipids (SP), 118 fatty acids (FA), 50 sterols (ST), and 2 prenol lipids (PR) (Figure 2A). Principal component analysis (PCA) of pre- and post-exercise samples (10 biological replicates per group) revealed pronounced lipidomic shifts induced by exercise stress. In the AH vs. BH comparison, PC1 and PC2 accounted for 53.52% and 11.20% of the total variance, respectively, while in the AL vs. BL comparison, PC1 and PC2 explained 52.93% and 12.48% of the variance. To further maximize intergroup separation and identify differential lipids, Orthogonal Projections to Latent Structures–Discriminant Analysis (OPLS-DA) was employed, which addresses the insensitivity of variables with low correlation better than PCA. The OPLS-DA results showed clear distinctions between pre- and post-exercise samples in both high and low remodeling groups, with well-defined clustering patterns. The unsupervised correlation analysis was consistent with the PCA results, indicating high similarity among biological replicates within each group (Figure 2F,H). Based on the criteria of variable importance in projection (VIP) > 1 and *p* < 0.05, a total of 1037 differential lipids were identified. Among them, 528 differential lipids were detected between the AL and BL groups (236 upregulated and 292 downregulated), while 509 differential lipids were identified between the AH and BH groups (211 upregulated and 298 downregulated). To further explore metabolic alterations associated with exercise stress, the differential lipids were clustered using the k-means algorithm, resulting in nine distinct clusters (Figure 2K). Cluster analysis revealed that lipids in clusters 5 and 8 showed consistent and significant changes after exercise in both remodeling groups. These lipids were mainly composed of triglycerides and phosphatidylglycerols, which play essential regulatory roles in energy metabolism and serve as critical molecular bases for maintaining metabolic homeostasis. In addition, notable intergroup differences were observed in cluster 1 (dominated by phosphatidylserines, 24%) and cluster 7 (dominated by phosphatidylcholines, 28.4%). KEGG pathway enrichment analysis of the differential lipids (Figure 2L) showed that the BH vs. AH comparison involved enrichment in a greater number of metabolic pathways compared with the BL vs. AL comparison. Considering the functional attributes of lipid metabolism, these results suggest that horses with higher degrees of cardiac remodeling possess a stronger capacity for lipid mobilization in response to exercise stress.

### 3.3. Transcriptomic Analysis

**Figure 3:**
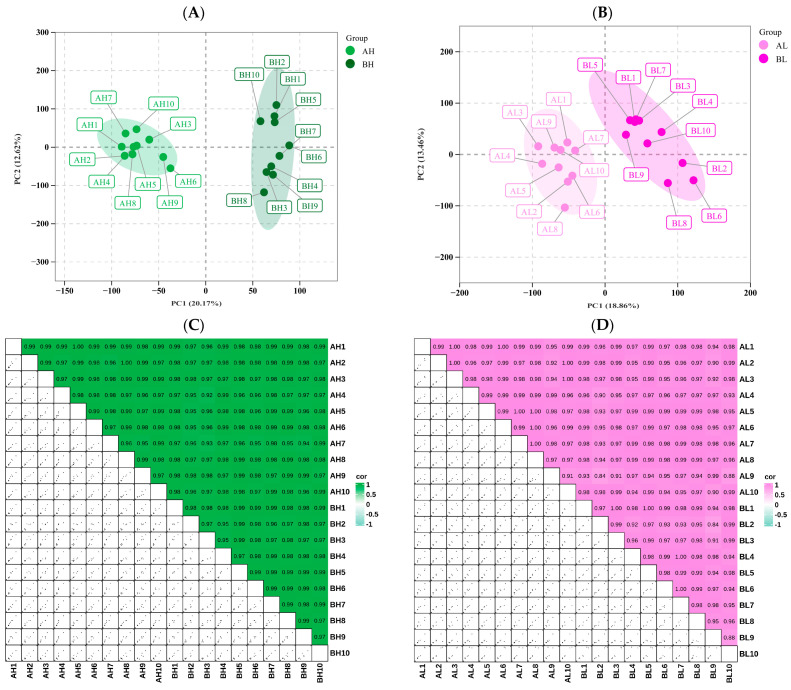

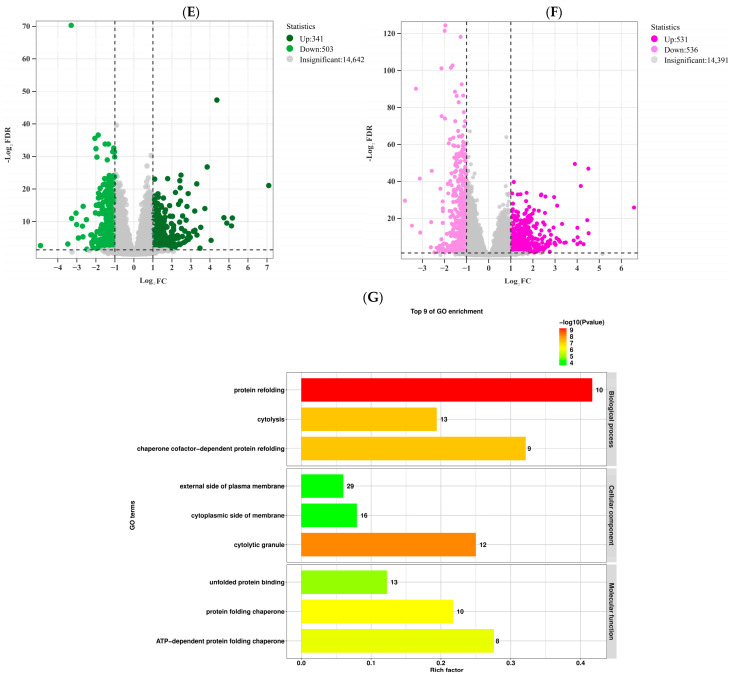

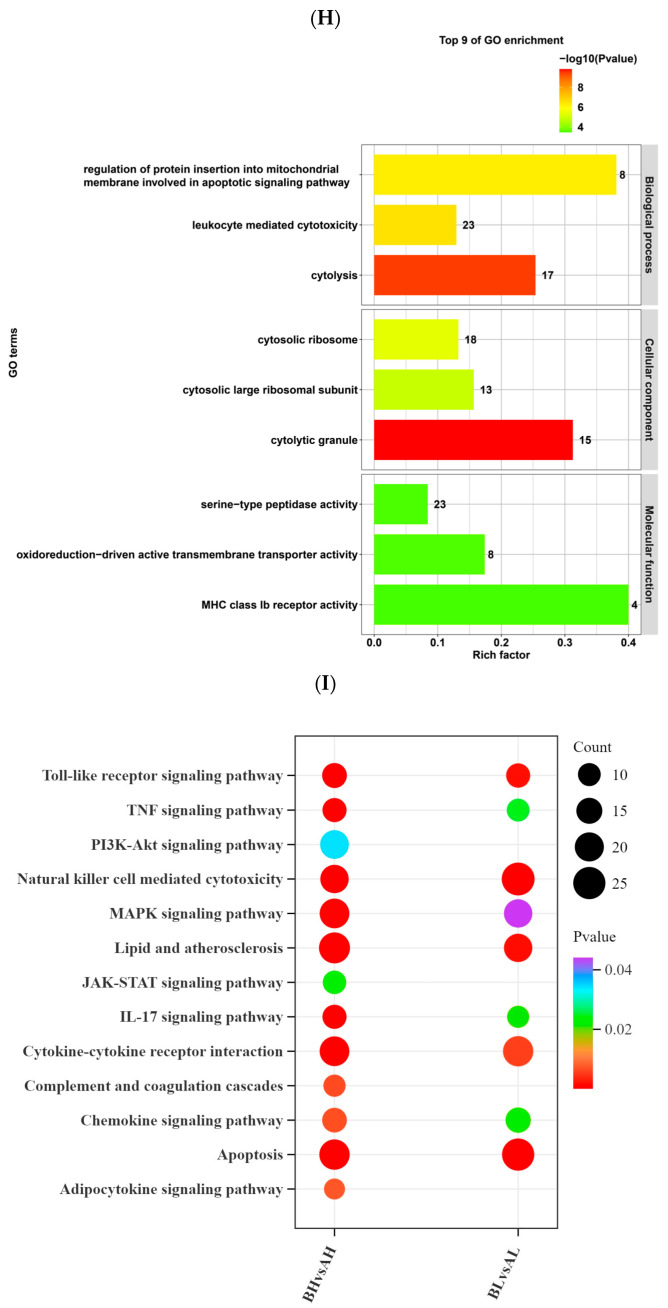
(**A**) PCA score plot of AH and BH groups. (**B**) PCA score plot of AL and BL groups. In the PCA plots, PC1 and PC2 represent the first and second principal components, respectively, and the percentages indicate the proportion of variance explained by each component. (**C**) Correlation matrix showing pairwise relationships among samples/metabolites in the AH vs. BH groups. (**D**) Correlation matrix showing pairwise relationships among samples/metabolites in the AL vs. BL groups. The upper right panels display correlation heatmaps of different variables, while the lower left panels show scatter plots of pairwise correlations. (**E**) Volcano plot of differentially expressed genes in AH vs. BH. (**F**) Volcano plot of differentially expressed genes in AL vs. BL. (**G**) GO enrichment bar plot for AH vs. BH. (**H**) GO enrichment bar plot for AL vs. BL. (**I**) KEGG pathway enrichment bubble plot of differentially expressed genes between pre- and post-exercise samples across groups. The *x*-axis represents the comparison groups, and the *y*-axis shows the enriched pathways.

To further investigate the transcriptional responses to exercise stress, blood samples collected from both groups of horses before and after the race were subjected to RNA sequencing, yielding a total of 28,548 genes. Principal component analysis (PCA) was performed to evaluate the overall expression patterns between the pre- and post-exercise groups. In the AH vs. BH comparison, PC1 and PC2 accounted for 20.17% and 12.62% of the total variance, respectively (Figure 3A), while in the AL vs. BL comparison, these components explained 18.86% and 13.46% of the total variance (Figure 3B). The unsupervised correlation analysis produced results consistent with the PCA, demonstrating a high degree of similarity among biological replicates within each group (Figure 3C,D). Based on the criteria of FDR < 0.05, *p* < 0.05, and fold change (FC) > 1, a total of 1911 differentially expressed mRNAs (DEmRNAs) were identified. Specifically, in the BL vs. AL comparison, 844 DEmRNAs were detected, including 341 upregulated and 503 downregulated genes. In the AH vs. BH comparison, 1067 DEmRNAs were identified, of which 531 were upregulated and 536 were downregulated. To further elucidate the transcriptional changes induced by exercise stress, Gene Ontology (GO) enrichment analysis was performed on the identified DEmRNAs. In the BH vs. AH comparison, significant enrichments were observed across all three GO categories. Biological Process: Notably enriched terms included protein refolding, cytolysis, and chaperone cofactor-dependent protein refolding. Cellular Component: Genes were significantly enriched in the external side of the plasma membrane, cytoplasmic side of the membrane, and cytolytic granule. Molecular Function: Prominent enrichments were detected for unfolded protein binding, protein folding chaperone activity, and ATP-dependent protein folding chaperone activity. These results indicate that processes related to protein folding, membrane-associated structures, and cytolytic activity may contribute to cardiac remodeling during the transition from pre- to post-competition (Figure 2G). In the BL vs. AL comparison, the biological process cytolysis exhibited the highest significance and a relatively high enrichment factor, suggesting its potential role in exercise-induced cardiac adaptation. Leukocyte-mediated cytotoxicity exhibited the highest enrichment factor, while the regulation of protein insertion into the mitochondrial membrane involved in apoptotic signaling was also significantly enriched. Cellular Component: The cytolytic granule showed the greatest significance and a high enrichment factor. Additionally, the cytosolic ribosome and the cytosolic large ribosomal subunit were enriched. Molecular Function: Serine-type peptidase activity displayed a high enrichment factor. Oxidoreduction-driven active transmembrane transporter activity and MHC class Ib receptor activity were also enriched. These results suggest that processes related to cell lysis, leukocyte-mediated cytotoxicity, ribosomal structures, and peptidase/transmembrane transporter activities contribute to the biological changes observed during the transition from pre- to post-competition (Figure 2H).

KEGG pathway enrichment analysis was performed on the differentially expressed mRNAs (DEmRNAs) across groups before and after exercise (Figure 3I). In the BH vs. AH comparison, DEmRNAs were significantly enriched in pathways including Toll-like receptor, TNF, PI3K-Akt, natural killer cell-mediated cytotoxicity, and MAPK signaling. Among these, the PI3K-Akt pathway exhibited the highest significance, whereas the Toll-like receptor and natural killer cell-mediated cytotoxicity pathways involved the largest number of genes. In the BL vs. AL comparison, DEmRNAs were enriched in natural killer cell-mediated cytotoxicity, apoptosis, Toll-like receptor, MAPK, and chemokine signaling pathways. Natural killer cell-mediated cytotoxicity and apoptosis contained the greatest number of genes, while the TNF and chemokine signaling pathways showed higher significance.

### 3.4. miRNA Analysis

**Figure 4:**
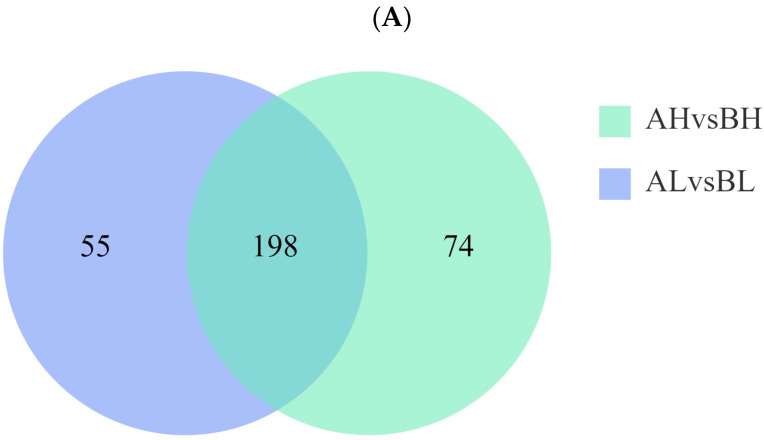

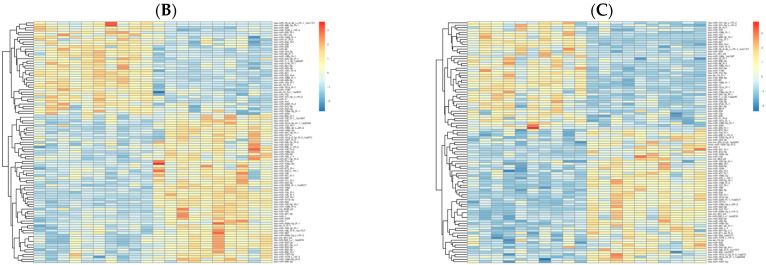

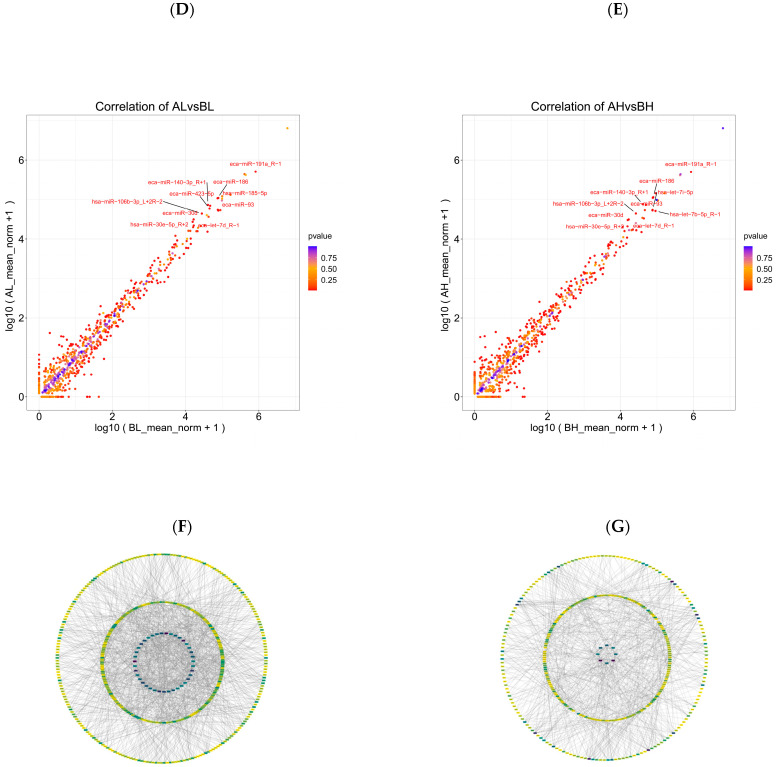
(**A**) Venn diagram showing shared and unique miRNAs between AH vs. BH and AL vs. BL groups. (**B**,**C**) Heatmaps of differentially expressed miRNAs (DEmiRNAs) in AH vs. BH and AL vs. BL, respectively. (**D**,**E**) Scatter plots of DEmiRNAs in AH vs. BH and AL vs. BL, with the top ten most significant DEmiRNAs labeled. (**F**,**G**) Networks of negative correlations between DEmiRNAs and differentially expressed genes (DEGs) for AH vs. BH and AL vs. BL, respectively.

To further investigate post-transcriptional regulatory responses to exercise, miRNA profiling was performed on pre- and post-exercise blood samples from both groups. Based on the thresholds of *p* < 0.05 and fold change (FC) > 1, a total of 198 miRNAs were shared between the two groups (Figure 4A). The unsupervised correlation analysis was consistent with the PCA results, showing a high degree of similarity among biological replicates within the same group (Figure 4B,C). In the AL vs. BL comparison, 253 differentially expressed miRNAs (DEmiRNAs) were identified, including 120 upregulated and 133 downregulated miRNAs. In the AH vs. BH comparison, 272 DEmiRNAs were detected, with 137 upregulated and 135 downregulated (Figure 4D,E). Target prediction was performed using TargetScan (v5.0) and miRanda (v3.3a) databases, and the predicted targets were cross-referenced with mRNAs obtained from RNA-seq data. In the AH vs. BH group, a total of 1276 miRNA–mRNA interaction pairs were identified, with eca-miR-186, eca-miR-23a, and eca-miR-23b serving as core miRNAs, exhibiting the highest number of connections. In the AL vs. BL group, 741 miRNA–mRNA pairs were detected, with eca-miR-486-3p, eca-miR-20b, and eca-miR-186 identified as the core miRNAs. The full list of miRNA–mRNA interactions is provided in Supplementary Table S2.

### 3.5. Integrated Transcriptomic and Metabolomic Analysis

**Figure 5:**
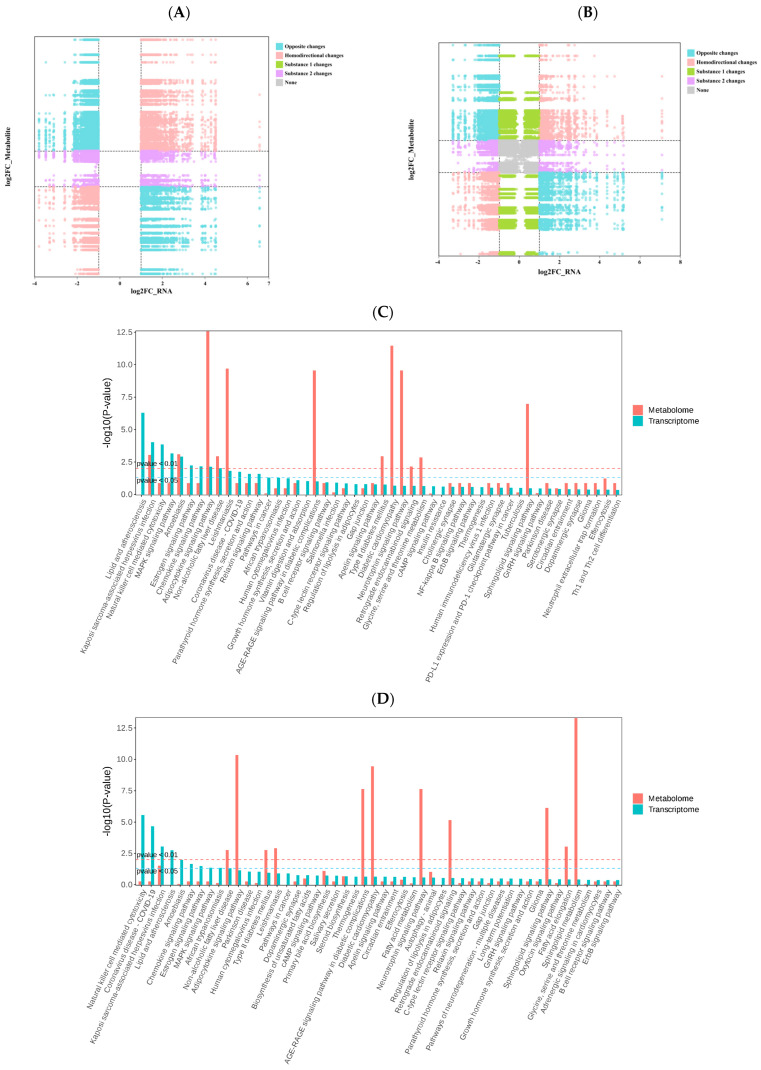

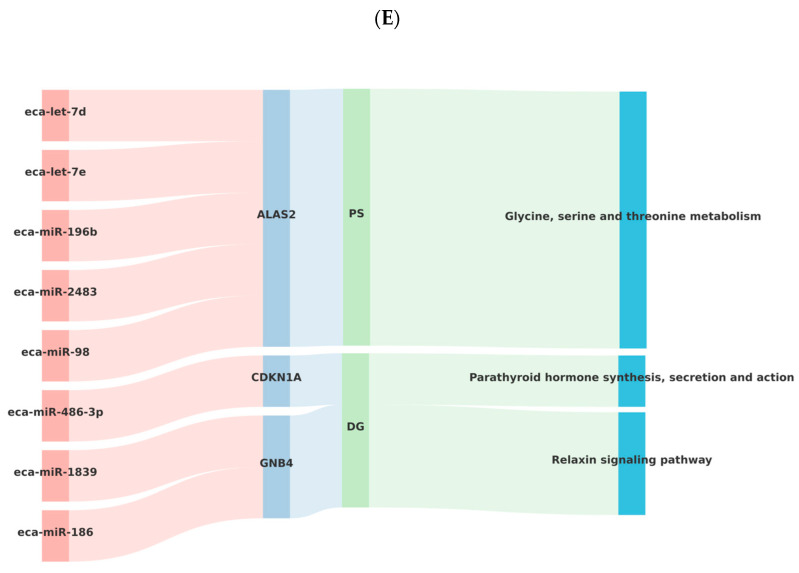
(**A**,**B**) Molecular expression and metabolite correlation analysis nine-quadrant plot. (**C**,**D**) The co-enrichment map of differentially expressed genes and metabolic pathways in the high cardiac remodeling group before and after the competition. (**E**) Integrate mRNA, miRNA, DEMs, and conduct comprehensive analysis using Payhway’s integrated Sangkey figure.

To elucidate the biological pathways underlying exercise-induced responses in horses with different degrees of cardiac remodeling, we performed an integrated analysis of transcriptomic and metabolomic data. Pearson correlation analysis was conducted between DEmRNAs and differential metabolites (DEMs) in the AH vs. BH and AL vs. BL groups (Figure 5A). Pairs with a correlation coefficient greater than 0.8 or less than −0.8 and *p* < 0.05 were considered significant, reflecting the strength of the transcript–metabolite associations (Figure 5B). Subsequently, KEGG pathway analysis was used to integrate transcriptomic and metabolomic results. The analysis revealed that both groups exhibited significant enrichment in the Sphingolipid signaling pathway, AMPK signaling pathway, and MAPK signaling pathway, suggesting that these pathways represent shared core regulatory mechanisms. In addition, AH vs. BH uniquely showed joint enrichment of DEGs and DEMs in pathways such as Glycine, serine and threonine metabolism, Glycosylphosphatidylinositol (GPI)-anchor biosynthesis, and Parathyroid hormone synthesis, secretion and action. Conversely, in AL vs. BL, DEGs and DEMs were specifically enriched in alpha-Linolenic acid metabolism, Arachidonic acid metabolism, and Fatty acid degradation pathways (Figure 5C,D).

Focusing on the AH vs. BH group, we further integrated miRNA, mRNA, and metabolite data to explore the underlying mechanisms that enable horses with high cardiac remodeling to maintain a stable and efficient energy regulation system during racing. Notably, differential genes and metabolites in AH vs. BH were uniquely enriched in the Glycine, serine and threonine metabolism pathway, which constitutes a core network connecting protein synthesis, energy supply, biomolecule biosynthesis, and oxidative protection. By integrating miRNA–mRNA interactions and evaluating correlations between DEGs and DEMs in the nine-quadrant plot, we constructed a Sankey diagram to visualize these multi-omics relationships (Figure 5E).

## 4. Discussion

While much of the existing literature has focused on collective changes before and after exercise or following long-term training, the present study emphasizes the dynamic coordination of molecular metabolism and physiological function in horses with superior cardiac structure and function during acute exercise at a controlled intensity. This approach provides a mechanistic understanding of how high-performance horses adapt to acute exercise stress and offers a theoretical basis for optimizing racehorse training programs to enhance their acute exercise resilience and competitive stability.

### 4.1. Differences in Cardiac Structure and Function of Yili Horses with Varying Degrees of Physiological Cardiac Remodeling

The physiological remodeling of the heart in response to exercise was first recognized in the late 19th century [15]. Subsequent studies have shown that the heart mass of athletes generally increases by 10–20%, and that these structural and functional changes are often reversible upon cessation of long-term training [16]. In the present study, echocardiographic measurements revealed that LVIDd in the BH group was 10.6% larger than in the BL group, while LVIDs was 7% larger, and LVM in BH exceeded BL by 34% (LVM (g) = 1.04 × [(LVIDd + LVFWd + IVSd)^3^ − LVIDd^3^] − 13.6). LVIDd reflects left ventricular size and diastolic function and is considered a typical adaptive feature of the “athlete’s heart” [17]. Consistent with these findings, a study comparing 1051 elite athletes with 338 sedentary controls reported significantly larger LVM, IVS, and LVID in athletes, with these parameters correlating with body composition. Similarly, in a study of endurance racehorses, elite horses exhibited markedly larger LVIDd, LVIDs, and LVM than non-elite horses, which aligns with the results of the present study. EDV reflects ventricular filling, and larger EDV in elite athletes allows for high stroke volume (SV) early in exercise without excessive heart rate elevation, delaying cardiac fatigue and supporting sustained high-intensity performance [18]. ESV, influenced by afterload and myocardial contractility, typically decreases by 15–30% during exercise compared to resting values [19], the same research results have also been found in speed horses [20,21]. In this study, BH exhibited 17% higher ESV than BL, yet SV in BH was 28% higher, attributable to the greater EDV in this group. Ejection fraction (EF%), calculated as EF% = (EDV − ESV)/EDV, is a widely used indicator of cardiac contractile function. Higher EF% reflects stronger cardiac contractility, directly impacting SV and cardiac output (CO) and enabling more efficient blood delivery to organs and tissues, thereby enhancing exercise performance [22,23]. Overall, the results of this study indicate that most cardiac structural parameters differed significantly between the two groups, demonstrating clear adaptive remodeling in horses with higher degrees of physiological cardiac remodeling.

### 4.2. Differences in Metabolomic Responses to Exercise in Yili Horses with Varying Degrees of Physiological Cardiac Remodeling

Blood lipids, including free fatty acids (FFAs), triglycerides (TGs), high-density/low-density lipoproteins (HDL/LDL), and phospholipids, serve as fundamental components for maintaining cellular structural integrity and physiological homeostasis. They play indispensable roles in supporting orderly cellular processes and overall organismal function [24].

After classifying the differential lipids between the two groups using K-means clustering, a key observation emerged: Cluster 1 lipids showed minimal differences in expression levels between the two groups prior to exercise, whereas post-exercise changes exhibited opposite trends. Specifically, lipids in the high cardiac remodeling group (BH) were significantly elevated, whereas those in the low cardiac remodeling group (BL) decreased. Notably, glycerophospholipids (GPs) accounted for a high proportion (17.68%) in this cluster, suggesting that BH horses may possess enhanced fatty acid oxidation capacity and more efficient lipid utilization pathways. Glycerophospholipids, as major constituents of cell membranes, also serve as important energy storage molecules and precursors for signaling pathways [25]. During exercise stress, the heart’s energy demand increases substantially. Horses in the BH group may have a more developed mitochondrial network and oxidative enzyme system, enabling more efficient uptake of fatty acids from the blood and their conversion into energy to support myocardial contraction and repair following high-intensity exercise [26]. In contrast, BL horses may lack comparable adaptive capacity; under acute exercise stress, they are likely to experience an energy supply-demand imbalance, with reduced ability to mobilize and oxidize fatty acids, resulting in relative energy substrate insufficiency.

Based on KEGG pathway analysis, we found that lipids in BH vs. AH were significantly enriched in the Sphingolipid signaling pathway and Sphingolipid metabolism. Sphingolipids are essential cell membrane components and key bioactive molecules involved in multiple cellular processes (e.g., cell growth, differentiation, apoptosis) and cardiac function regulation. Their metabolic products, such as ceramide, sphingosine, and sphingosine-1-phosphate (S1P), play critical signaling roles in both physiological and pathological cardiac contexts. Post acute exercise, sphingolipid enrichment may reflect cardiomyocyte membrane remodeling as an adaptive response to exercise-induced stress, which enhances cellular resilience, repairs damage, and optimizes membrane-associated protein function. Ceramide is generally recognized as a pro-apoptotic molecule, whereas S1P promotes cell survival and proliferation; these two metabolites are involved in exercise-induced cardiac remodeling—a complex process characterized by adaptive cardiomyocyte growth while avoiding pathological apoptosis [27]. The enrichment of this pathway suggests that the heart is actively and dynamically regulating these processes to maintain functional homeostasis during acute exercise.

### 4.3. Transcriptomic Differences in Yili Horses with Varying Degrees of Physiological Cardiac Remodeling Before and After Exercise

Acute exercise has been shown to induce changes in the levels of multiple miRNAs in blood and specific tissues, such as adipose tissue and saliva. In the BH vs. AH group, the miRNA–mRNA interaction network revealed that miR-186, miR-23a, miR-23b, and members of the let-7 family exhibited the highest connectivity. Previous studies have reported that serum exosomal miR-186-5p is significantly elevated in patients with acute myocardial infarction (AMI) and coronary artery disease, positively correlating with lipid levels, the degree of coronary stenosis, and the risk of major adverse cardiovascular events (MACE) [28]. In this study, miR-186 was predicted to target GNB4 and RGS5. GNB4 is associated with atrial fibrillation and resting heart rate and is involved in cardiac electrophysiological regulation [29]. RGS5 modulates cardiac repolarization and electrical stability, with its loss leading to delayed ventricular repolarization and spatial heterogeneity [30]. miR-23a has been reported to decrease during myocardial ischemia–reperfusion (I/R) injury, which promotes oxidative stress and apoptosis; conversely, its overexpression mitigates apoptosis by inhibiting FoxO3 and BIM expression [31]. miR-23b can target GATA6 to suppress IGF-1 expression, promoting cardiomyocyte apoptosis and inhibiting proliferation. The let-7 family, one of the earliest discovered microRNA families, plays important roles in cardiac development, function maintenance, and disease processes, and emerging evidence suggests roles in exercise adaptation and skeletal muscle regulation [32]. Let-7 family members are highly expressed in the cardiovascular system, contributing to the differentiation of embryonic stem cells into cardiovascular lineages and promoting cardiomyocyte maturation [33]. Specifically, let-7a can suppress pathological cardiac hypertrophy by targeting calmodulin. In this study, eca-let-7d and eca-let-7e were predicted to target ALAS2, a key enzyme involved in oxygen transport and hypoxic stress regulation. By modulating the heme biosynthesis pathway, ALAS2 plays a crucial role in cardiomyocyte development and function, enhancing cellular adaptation to hypoxic conditions [34].

In the BL vs. AL group, highly connected miRNAs included miR-486-3p, miR-20b, and miR-24. miR-486-3p is enriched in muscle and upregulated in the heart post-exercise, mediating exercise-induced physiological cardiac growth and protecting against ischemia/reperfusion (I/R) injury [35]; notably, a study on equine plasma extracellular particle miRNA during endurance racing also highlighted miR-486-3p as a key regulator in exercise-related cardiac responses [36]. In the present study, miR-486 was predicted to target ATF3, which plays complex roles in cardiovascular pathogenesis (e.g., atherosclerosis, hypertrophy) and protection, underscoring its involvement in cardiac adaptation and stress responses [37].

### 4.4. Integrated Transcriptomic and Metabolomic Analysis of Yili Horses with Varying Degrees of Physiological Cardiac Remodeling

#### 4.4.1. Sphingolipid Metabolism and Sphingolipid Signaling Pathway

Both BH vs. AH and BL vs. AL groups showed significant enrichment in these two pathways. As discussed in the metabolomic analysis, sphingolipid metabolism and signaling are crucial for membrane structure and cellular signaling. Their co-activation indicates that acute exercise induces changes in membrane fluidity and remodeling of cellular signaling networks in both high and low cardiac remodeling horses.

#### 4.4.2. Adipocytokine Signaling Pathway

The adipocytokine signaling pathway plays a central role in exercise-mediated regulation. Key molecules in this pathway, such as adiponectin, leptin, and TNF-α, rapidly respond to changes in energy demand by coordinating insulin sensitivity, fatty acid oxidation, and glucose metabolism [38].

#### 4.4.3. AMPK Signaling Pathway

Exercise is a major activator of the AMP-activated protein kinase (AMPK) pathway. During physical activity, the AMP/ATP ratio increases, leading to AMPK activation, which rapidly mobilizes glucose and fatty acids and promotes mitochondrial adaptations to meet the metabolic demands of skeletal muscle and the heart [39]. This pathway intersects with the adipocytokine signaling pathway: adiponectin activates AMPK to enhance fatty acid oxidation and insulin sensitivity, while AMPK suppresses pro-inflammatory factors such as TNF-α [40]. This coordinated mechanism plays a key role in exercise-induced metabolic reprogramming and inflammation balance. Furthermore, studies have shown that total AMPK activity in the heart increases during exercise, with a more pronounced rise under high-intensity conditions [41].

#### 4.4.4. Fatty Acid Elongation

Fatty acid elongation is a key metabolic process responsible for the synthesis of long-chain (LCFA) and very-long-chain fatty acids (VLCFA), primarily occurring in the endoplasmic reticulum. This pathway extends carbon chains by two-carbon units through a series of four enzymatic reactions, which is essential for maintaining cell membrane structure, energy storage, and signal transduction. The AMPK signaling pathway can regulate fatty acid synthesis, oxidation, and elongation, thereby contributing to cellular energy homeostasis.

These energy metabolism and lipid remodeling pathways were significantly enriched in both groups of horses before and after exercise, suggesting common mechanisms underlying acute exercise adaptation in high- and low-cardiac remodeling horses. However, notable differences between the groups were also observed.

#### 4.4.5. Alpha-Linolenic Acid, Arachidonic Acid, and Linoleic Acid Metabolism

During exercise, the body requires substantial energy to sustain muscle contraction and physiological functions. Fatty acids, including ALA, AA, and LA, serve as important energy sources, especially during prolonged low-to-moderate intensity exercise. These fatty acids undergo β-oxidation to generate ATP, providing energy for muscle activity [42].

#### 4.4.6. Glycine, Serine, and Threonine Metabolism

In this study, the glycine, serine, and threonine metabolism pathway was specifically activated in the high-cardiac remodeling group [43]. Glycine contributes to the synthesis of purines and creatine, both of which are critical for energy metabolism. Creatine acts as a rapid energy buffer in muscle cells, particularly during high-intensity exercise. Serine participates in amino acid transport, ensuring sufficient availability for muscle protein synthesis and repair [44], while threonine can be converted to α-ketobutyrate, entering the TCA cycle to provide energy [45]. Transcriptomic analysis indicated that eca-let-7d, eca-let-7e, eca-miR-196b, eca-miR-2483, and eca-miR-98 target ALAS2 and, together with GCSH, are enriched in this pathway. Metabolomic analysis further showed that phosphatidylserine species such as PS(17:0/16:1), PS(18:0/18:1), and PS(20:0/18:1) are enriched within the pathway. These metabolites collectively participate in glycine, serine, and threonine metabolism, influencing energy supply, inflammatory responses, and muscle function during exercise, thereby highlighting the molecular mechanisms by which the high-cardiac remodeling group maintains energy homeostasis and myocardial protection under acute exercise stress.

#### 4.4.7. Limitations Statement

Currently, studies investigating whether changes in mRNA, miRNA, and metabolites in blood samples can accurately reflect the specific molecular mechanisms in cardiac tissues (especially cardiomyocytes or cardiac fibroblasts) remain relatively limited, which is one of the main limitations of this study.

## 5. Conclusions

This study employed an integrated multi-omics approach to elucidate the differential molecular responses of horses with varying degrees of cardiac remodeling under acute exercise. The results indicate that horses in the high-cardiac remodeling group exhibit more favorable structural and functional adaptations to exercise. Specifically, eca-let-7d, eca-let-7e, eca-miR-196b, eca-miR-2483, and eca-miR-98 target ALAS2; together with phosphatidylserine molecules such as PS(17:0/16:1), PS(18:0/18:1), and PS(20:0/18:1), these molecules may be involved in processes related to energy supply efficiency, inflammatory responses, and muscle function during exercise via the glycine-serine-threonine metabolic pathway. These findings provide novel insights into the individual variability of exercise-induced cardiac adaptations and offer potential molecular targets for the precise assessment of equine athletic performance and the optimization of training strategies.

## Data Availability

The original contributions presented in the study are included in the article/Supplementary Material, further inquiries can be directed to the corresponding authors.

## Supplementary Materials

The following supporting information can be downloaded at: https://www.mdpi.com/article/10.3390/ani15223251/s1, Supplement Text S1;Echocardiography acquisition method. Supplement Text S2: Sample QC Report. Supplement Text S3: Sample collection and preparation. Supplement Text S4; miRNA bioinformatics analysis method. Supplement Text S5: TruSeq Small RNA Sample Preparation. Supplement Table S1: RNA integrity. Supplement Table S2: miRNA-mRNA negative regulatory pairs.

## References

[1] De Innocentiis C., Ricci F., Khanji M.Y., Aung N., Tana C., Verrengia E., Petersen S.E., Gallina S. (2018). Athlete’s heart: Diagnostic challenges and future perspectives. Sports Med..

[2] Klein D.J., Anthony T.G., McKeever K.H. (2021). Metabolomics in equine sport and exercise. J. Anim. Physiol. Anim. Nutr..

[3] Heaney L.M., Deighton K., Suzuki T. (2017). Non-targeted metabolomics in sport and exercise science. J. Sports Sci..

[4] Nieman D.C., Shanely R.A., Luo B., Meaney M.P., Dew D.A., Pappan K.L. (2014). Metabolomics approach to assessing plasma 13- and 9-hydroxy-octadecadienoic acid and linoleic acid metabolite responses to 75-km cycling. Am. J. Physiol. Regul. Integr. Comp. Physiol..

[5] Pohjanen E., Thysell E., Jonsson P., Eklund C., Silfver A., Carlsson I.B., Lundgren K., Moritz T., Svensson M.B., Antti H. (2007). A multivariate screening strategy for investigating metabolic effects of strenuous physical exercise in human serum. J. Proteome Res..

[6] Eyileten C., Wicik Z., Fitas A., Marszalek M., Simon J.E., De Rosa S., Wiecha S., Palatini J., Postula M., Malek L. (2022). Altered circulating microRNA profiles after endurance training: A cohort study of ultramarathon runners. Front. Physiol..

[7] Liu X., Platt C., Rosenzweig A. (2017). The role of MicroRNAs in the cardiac response to exercise. Cold Spring Harb. Perspect. Med..

[8] Drown M., Crawford D., Oleksiak M. (2022). mRNA expression explains metabolic and thermal physiology. bioRxiv.

[9] Gao C., Wang Y. (2020). mRNA metabolism in cardiac development and disease: Life after transcription. Physiol. Rev..

[10] Yuan T., Wu M., Zhu C., Yu H., Pham M.D., Bottermann K., Mao Y., Langner M., Peitzsch M., Das A.P. (2024). Combinatorial miRNA1a/15b interference drives adult cardiac regeneration. medRxiv.

[11] Fulghum K. (2022). Metabolic Foundations of Exercise-Induced Cardiac Growth. Ph.D. Thesis.

[12] Wang T., Yang X., Zeng Y., Wang J., Li X., Shen Z., Liu H., Zhang Y., Chen W., Xu L. (2025). Integrating miRNA, mRNA, and Targeted Metabolomics Analyses to Explore the Regulatory Mechanism of Cardiac Remodeling in Yili Horses. Biology.

[13] Ringmark S., Lindholm A., Hedenström U., Lindinger M., Dahlborn K., Kvart C., Jansson A. (2015). Reduced high intensity training distance had no effect on VLa4 but attenuated heart rate response in 2–3-year-old Standardbred horses. Acta Vet. Scand..

[14] Wang T., Meng J., Peng X., Huang J., Huang Y., Yuan X., Li K., Chen W., Zhang L., Liu M. (2025). Metabolomics analysis and mRNA/miRNA profiling reveal potential cardiac regulatory mechanisms in Yili racehorses under different training regimens. PLoS ONE.

[15] Pelliccia A., Maron B.J., De Luca R., Di Paolo F.M., Spataro A., Culasso F. (2002). Remodeling of left ventricular hypertrophy in elite athletes after long-term deconditioning. Circulation.

[16] Baggish A.L., Wang F., Weiner R.B., Elinoff J.M., Tournoux F., Boland A., Picard M.H., Hutter A.M., Wood M.J. (2008). Training-specific changes in cardiac structure and function: A prospective and longitudinal assessment of competitive athletes. J. Appl. Physiol..

[17] Choi N.S., Jung I.W., Kang H.S., Cho C.W., Kim K.S., Kim M.S., Song J.S., Bae J.H. (1996). Echocardiographic Evaluation of Left Ventricle before and after Maximum Exercise in Track Athletes. J. Korean Soc. Echocardiogr..

[18] Stöhr E.J., González-Alonso J., Shave R. (2011). Left ventricular mechanical limitations to stroke volume in healthy humans during incremental exercise. Am. J. Physiol.-Heart Circ. Physiol..

[19] Treibert J., Friederich J., Fischer S., Küchenhoff H., Wess G. (2024). Reference intervals for various measurements of canine left atrial size and function obtained using two-dimensional and three-dimensional echocardiography. J. Vet. Cardiol..

[20] Wang T., Meng J., Yang X., Zeng Y., Yao X., Ren W. (2025). Differential Metabolomics and Cardiac Function in Trained vs. Untrained Yili Performance Horses. Animals.

[21] Wang T., Meng J., Wang J., Ren W., Yang X., Adina W., Bao Y., Zeng Y., Yao X. (2025). Absolute Quantitative Lipidomics Reveals Differences in Lipid Compounds in the Blood of Trained and Untrained Yili Horses. Vet. Sci..

[22] Tsougos E., Angelidis G., Gialafos E., Tzavara C., Tzifos V., Tsougos I., Georgoulias P. (2018). Myocardial strain may predict exercise tolerance in patients with reduced and mid-range ejection fraction. Hell. J. Cardiol..

[23] Grenacher P.A., Schwarzwald C.C. (2010). Assessment of left ventricular size and function in horses using anatomical M-mode echocardiography. J. Vet. Cardiol..

[24] Cockcroft S. (2021). Mammalian lipids: Structure, synthesis and function. Essays Biochem..

[25] Cui S.F., Li W., Niu J., Zhang C.Y., Chen X., Ma J.Z. (2015). Acute responses of circulating microRNAs to low-volume sprint interval cycling. Front. Physiol..

[26] Da Dalt L., Cabodevilla A.G., Goldberg I.J., Norata G.D., Giorgino T., Quagliarini F. (2023). Cardiac lipid metabolism, mitochondrial function, and heart failure. Cardiovasc. Res..

[27] Foran D., Antoniades C., Akoumianakis I. (2024). Emerging roles for sphingolipids in cardiometabolic disease: A rational therapeutic target?. Nutrients.

[28] Li Q., Wu M., Fang G., Li K., Cui W., Li L., Li X., Wang J., Cang J. (2021). MicroRNA-186-5p downregulation inhibits osteoarthritis development by targeting MAPK1. Mol. Med. Rep..

[29] Patel K.K., Venkatesan C., Abdelhalim H., Zeeshan S., Arima Y., Linna-Kuosmanen S., Ahmed Z. (2023). Genomic approaches to identify and investigate genes associated with atrial fibrillation and heart failure susceptibility. Hum. Genom..

[30] Song Z., Liu Y., Liu X., Qin M. (2021). Absence of Rgs5 influences the spatial and temporal fluctuation of cardiac repolarization in mice. Front. Physiol..

[31] Yang Q.H., Yang M., Zhang L.L., Xiao M.C., Zhao Y., Yan D.X. (2017). The mechanism of miR-23a in regulating myocardial cell apoptosis through targeting FoxO3. Eur. Rev. Med. Pharmacol. Sci..

[32] Smith A.S.T., Macadangdang J., Leung W., Laflamme M.A., Kim D.-H. (2017). Human iPSC-derived cardiomyocytes and tissue engineering strategies for disease modeling and drug screening. Biotechnol. Adv..

[33] Gan J., Yuan J., Liu Y., Lu Z., Xue Y., Shi L., Zeng H. (2020). Circular RNA_101237 mediates anoxia/reoxygenation injury by targeting let-7a-5p/IGF2BP3 in cardiomyocytes. Int. J. Mol. Med..

[34] He Y., Wang X., Li D., Zhu Q., Xiang Y., He Y., Zhang H. (2024). ALAS2 overexpression alleviates oxidative stress-induced ferroptosis in aortic aneurysms via GATA1 activation. J. Thorac. Dis..

[35] Bei Y., Lu D., Meng X., Zhu Y., Liang X., Xiao J. (2019). P5399 microRNA-486 mediates exercise-induced cardiac growth and prevents cardiac ischemia-reperfusion injury. Eur. Heart J..

[36] de Oliveira G.P., Porto W.F., Palu C.C., Pereira L.M., Reis A.M.M., Marçola T.G., Teixeira-Neto A.R., Franco O.L., Pereira R.W. (2021). Effects of endurance racing on horse plasma extracellular particle miRNA. Equine Vet. J..

[37] Ke H., Chen Z., Zhao X., Yang C., Luo T., Ou W., Wang L., Liu H. (2023). Research progress on activation transcription factor 3: A promising cardioprotective molecule. Life Sci..

[38] Webb R., Hughes M.G., Thomas A.W., Morris K. (2017). The ability of exercise-associated oxidative stress to trigger redox-sensitive signalling responses. Antioxidants.

[39] Behera S., Ghosh Roy A. (2024). Exercise-induced differential transcriptional output of AMPK signalling improves axon regeneration and functional recovery. bioRxiv.

[40] An H., Jang Y., Choi J., Hur J., Kim S., Kwon Y. (2024). New insights into AMPK, as a potential therapeutic target in metabolic dysfunction-associated steatotic liver disease and hepatic fibrosis. Biomol. Ther..

[41] Coven D.L., Hu X., Cong L., Bergeron R., Shulman G.I., Hardie D.G., Young L.H. (2003). Physiological role of AMP-activated protein kinase in the heart: Graded activation during exercise. Am. J. Physiol.-Endocrinol. Metab..

[42] Fang C., Pan J., Qu N., Lei Y., Han J., Zhang J., Han D. (2022). The AMPK pathway in fatty liver disease. Front. Physiol..

[43] Huang S., Shangguan R., Chen S., Lai X., Han H., Sun J. (2025). Mechanism of Fatty Acid Metabolism and Regulation by Lactate During Exercise in White Adipose and Skeletal Muscle Tissue: A Review. Sports Med.-Open.

[44] Pranzini E., Muccillo L., Nesi I., Santi A., Mancini C., Lori G., Genovese M., Lottini T., Comito G., Caselli A. (2024). Limiting serine availability during tumor progression promotes muscle wasting in cancer cachexia. Cell Death Discov..

[45] Cao H., Zha C., Shao F., Wang L., Tan B. (2020). Amino acids regulate energy utilization through mammalian target of rapamycin complex 1 and adenosine monophosphate activated protein kinase pathway in porcine enterocytes. Anim. Nutr..

