# Supplementary material for: Multi-Omics Deciphers Divergent Mechanisms in Differentially Cardiac-Remodeled Yili Horses Under Conditions of Equivalent Power Output
Source: Animals (Basel). 2025 Nov 9;15(22):3251. doi: 10.3390/ani15223251 (PMC12649268; doi:10.3390/ani15223251)
Supplement: Supplementary file 1 [file animals-15-03251-s001.zip › Supplement Text S1.pdf]

Supplement Text S1

Prior to imaging, horses were secured on a dedicated restraint platform and allowed time to settle. Body height was measured using a measuring rod, and body weight was recorded with a scale. The right parasternal region was cleaned, and a 2.5 MHz probe was used to obtain ultrasound images, with the maximum imaging depth set at 30 cm and the sector angle at 110°. Heart rate was maintained within 32–45 beats per minute, corresponding to the resting range.

All ultrasound examinations were performed by the same experienced operator, with three independent imaging sessions conducted to ensure reproducibility. Acquired images included right parasternal long-axis B-mode, right parasternal left ventricular outflow tract B-mode, and right parasternal short-axis B- and M-mode images, with both static and dynamic images captured at end-diastole and end-systole. A total of 22 cardiac parameters were measured: right ventricular end-diastolic diameter (RVDd), interventricular septum end-diastolic thickness (IVSd), left ventricular end-diastolic diameter (LVIDd), left ventricular free wall end-diastolic thickness (LVFWd), right ventricular end-systolic diameter (RVDs), interventricular septum end-systolic thickness (IVSs), left ventricular end-systolic diameter (LVIDs), left ventricular free wall end-systolic thickness (LVFWs), left ventricular minor axis diameter (LVminor), mitral valve annulus diameter (MVD), left atrial end-diastolic diameter (LADd), left atrial end-systolic diameter (LADs), aortic root end-diastolic diameter (AODd), pulmonary artery end-diastolic diameter (PAd), aortic root end-systolic diameter (AODs), pulmonary artery end-systolic diameter (PAs), left ventricular end-diastolic volume (EDV), left ventricular end-systolic volume (ESV), ejection fraction (EF%), stroke volume (SV), fractional shortening (FS%), and left ventricular mass (LVM). For subsequent analyses, the mean value of the three independent measurements was used.
