# Supplementary material for: Multi-Omics Deciphers Divergent Mechanisms in Differentially Cardiac-Remodeled Yili Horses Under Conditions of Equivalent Power Output
Source: Animals (Basel). 2025 Nov 9;15(22):3251. doi: 10.3390/ani15223251 (PMC12649268; doi:10.3390/ani15223251)
Supplement: Supplementary file 1 [file animals-15-03251-s001.zip › Supplement Text S3.pdf]

I. Sample collection and preparation

1. RNA extraction and detection

Plant samples were extracted by ethanol precipitation and CTAB-PBIOZOL. Animal samples were extracted by Trizol method. After successful extraction, RNA was dissolved by adding 50 μL of DEPC-treated water. Subsequently, total RNA was identified and quantified using a Qubit fluorescence quantifier and a Qsep400 high-throughput biofragment analyzer.

2. mRNA library construction

1) By utilizing the structural characteristic that most eukaryotic mRNAs carry a polyA tail, mRNAs with polyA tails were enriched by Oligo(dT) magnetic beads;

2) The purified mRNAs were cleaved into small fragments with fragmentation buffer at a suitable temperature;

3) First-strand cDNAs were produced by reverse transcription using a random hexamer primer;

4) Second-strand cDNAs are synthesized (**strand-specific library: dUTPs were used instead of dTTPs in the second-strand synthesis to incorporate dUTPs in the second-strand cDNAs, while the high-fidelity DNA polymerase used in this method could not amplify uracil-containing DNA templates, thus realizing the strand-specificity**), while simultaneously performing end repair and dA-Tailing;

5) Sequencing adapter ligation was performed, followed by DNA magnetic bead purification and fragment selection after ligation was completed to yield a library with 250-350 bp insert fragments;

6) The ligated products were amplified by PCR and purified again using DNA magnetic beads, where the products were solubilized with nuclear-free water;

7) After the initial library was constructed, a Qubit fluorescence quantifier was used for concentration detection, followed by a Qsep400 high-throughput biofragment analyzer for fragment size detection.

3.Sequencing run

After passing the library check, the different libraries were sequenced in Illumina after pooling them according to the effective concentration and the target sequencing output data volume, yielding 150bp paired-end reads. The basic principle of sequencing is to synthesize and sequence at the same time. Four types of fluorescently labeled dNTPs, DNA polymerase, and junction primers were added to the sequencing flow cell for amplification. When extending the complementary strand of each sequencing cluster, each fluorescently labeled dNTP added emits corresponding fluorescence, and the sequencer captures the fluorescence signals, and converts the light signals into sequencing peaks through computer software, so as to obtain the sequence information of the fragment to be tested.

II. Data Analysis

1. Data Quality Control

Data quality control was performed using fastp to remove reads with adapters. Paired reads were removed under the following conditions: when the the number of N in any sequencing read exceeded 10% of the length of that read, and when any sequencing read contained low-quality bases (Q<=20) exceeding 50% of the length of that read. Subsequent analyses were based on clean reads.

2. Sequence Alignment to the Reference Genome

The reference genome and its annotation files were downloaded from a specified website. HISAT was used to build an index, and clean reads were aligned to the reference genome.

3. Prediction of Novel Transcripts

Novel gene prediction was performed using StringTie, which utilizes network flow algorithms and optional de novo transcript assembly to splice transcripts. Compared to software like Cufflinks, StringTie can assemble more complete and accurate transcripts and is faster in the splicing process.

4. Quantification of Gene Expression Levels

Gene expression levels were quantified using featureCounts to calculate gene alignment statistics. Subsequently, FPKM (Fragments Per Kilobase Million) values for each gene were computed based on gene length. FPKM is currently the most used method for estimating gene expression levels.

5. Differential Analysis

DESeq2 was used for differential gene expression analysis between two groups, and Benjamini & Hochberg correction was applied to P-values. Corrected P-values and log2 fold change were used as thresholds for significant differential expression.

6. Differential Gene Enrichment Analysis

Enrichment analysis was performed based on the hypergeometric test, with pathway-based hypergeometric distribution testing for KEGG and GO term-based analysis for GO.

7. Differential Splicing Analysis

rMATS was used to analyze alternative splicing events, including SE (skipped exon), RI (retained intron), MXE (mutually exclusive exons), A5SS (alternative 5' splice site), and A3SS (alternative 3' splice site).

8. SNP Analysis

GATK was used for variant site analysis, and annovar was used for variant annotation.

9. Differential Gene-Protein Interaction Analysis

Analysis of protein interactions for differentially expressed genes was based on the STRING database, which contains known and predicted protein-protein interactions. For species present in the database, a network was constructed by extracting the target gene list from the database. Otherwise, blastx was used to align target gene sequences with selected reference protein sequences, and a network was established based on known interactions in the selected reference species.

10. Gene Set Enrichment Analysis (GSEA)

GSEA was conducted using the gsea tool for gene set enrichment analysis.

11. Weighted Gene Co-expression Network Analysis (WGCNA)

WGCNA was performed for weighted gene co-expression network analysis.
