# Supplementary material for: Multi-Omics Deciphers Divergent Mechanisms in Differentially Cardiac-Remodeled Yili Horses Under Conditions of Equivalent Power Output
Source: Animals (Basel). 2025 Nov 9;15(22):3251. doi: 10.3390/ani15223251 (PMC12649268; doi:10.3390/ani15223251)
Supplement: Supplementary file 1 [file animals-15-03251-s001.zip › Supplement Text S4.pdf]

miRNA bioinformatics analysis method

Raw reads were subjected to an in-house program, ACGT101-miR (v4.2) to remove adapter dimers, junk, low complexity, common RNA families (rRNA, tRNA, snRNA, snoRNA) and repeats. Subsequently, unique sequences with length in 18~26 nucleotide were mapped to specific species precursors in miRBase 22.1 by BLAST search to identify known miRNAs and novel 3p- and 5p- derived miRNAs. Length variation at both 3’ and 5’ ends and one mismatch inside of the sequence were allowed in the alignment. The unique sequences mapping to specific species mature miRNAs in hairpin arms were identified as known miRNAs. The unique sequences mapping to the other arm of known specific species precursor hairpin opposite to the annotated mature miRNA-containing arm were considered to be novel 5p- or 3p- derived miRNA candidates. The remaining sequences were mapped to other selected species precursors (with the exclusion of specific species) in miRBase 22.1 by BLAST search, and the mapped pre-miRNAs were further BLASTed against the specific species genomes to determine their genomic locations. The above two we defined as known miRNAs. The unmapped sequences were BLASTed against the specific genomes, and the hairpin RNA structures containing sequences were predicated from the flank 80nt sequences using RNAfold software (http://rna.tbi.univie.ac.at/cgi-bin/RNAfold.cgi). The criteria for secondary structure prediction were: (1) number of nucleotides in one bulge in stem (≤12), (2) number of base pairs in the stem region of the predicted hairpin (≥16), (3) cutoff of free energy (kCal/mol ≤ -15), (4) length of hairpin (up and down stems + terminal loop ≥50), (5) length of hairpin loop (≤20), (6) number of nucleotides in one bulge in mature region (≤8), (7) number of biased errors in one bulge in mature region (≤4), (8) number of biased bulges in mature region (≤2), (9) number of errors in mature region (≤7), (10) number of base pairs in the mature region of the predicted hairpin (≥12), (11) percent of mature in stem (≥80).
