# Supplementary material for: Multi-Omics Deciphers Divergent Mechanisms in Differentially Cardiac-Remodeled Yili Horses Under Conditions of Equivalent Power Output
Source: Animals (Basel). 2025 Nov 9;15(22):3251. doi: 10.3390/ani15223251 (PMC12649268; doi:10.3390/ani15223251)
Supplement: Supplementary file 1 [file animals-15-03251-s001.zip › Supplement Text S5.pdf]

TruSeq Small RNA Sample Preparation

Experienced User Card

FOR RESEARCH USE ONLY

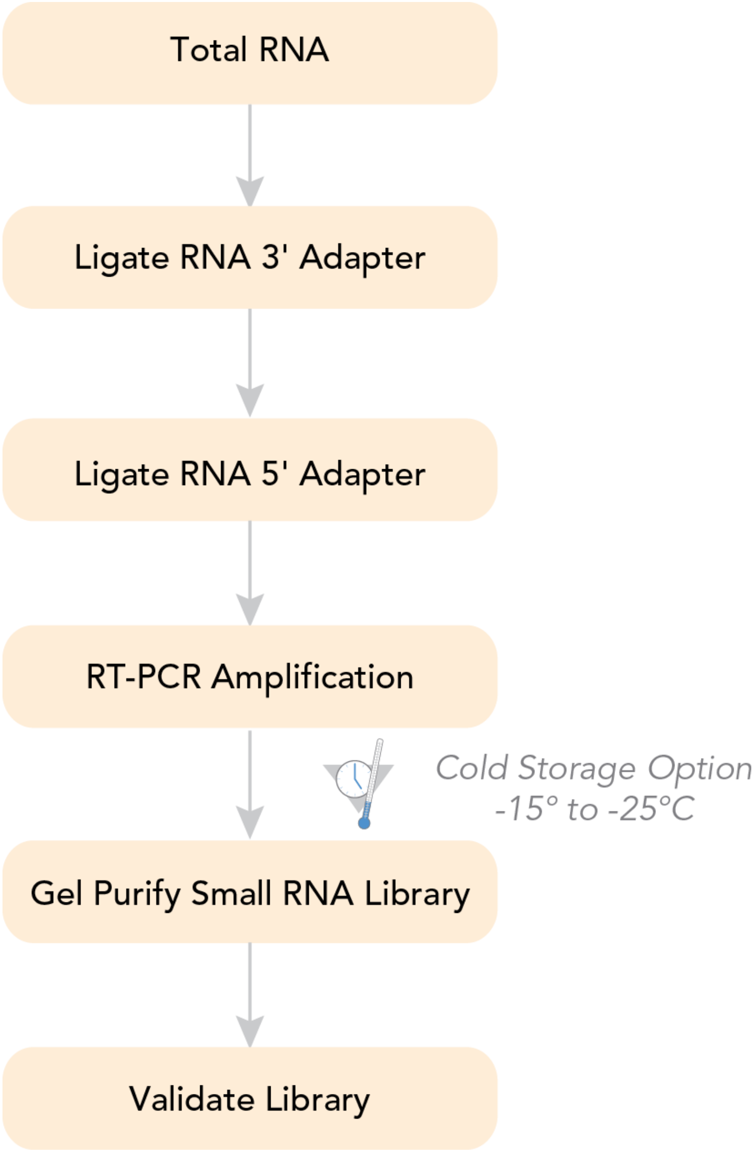

NOTE

- Unless familiar with the protocol in the latest version of the TruSeq Small RNA Sample Preparation Guide, new or less experienced users are strongly advised to follow the protocol in the guide before using this Experienced User Card.
- For optimal sample tracking and quality control, fill out the TruSeq Small RNA Sample Preparation Lab Tracking Form as you perform TruSeq Small RNA Sample Preparation.

Ligate 3' and 5' Adapters

This process describes the sequential ligation of the RNA 3' and RNA 5' RNA adapters to the sample.

Illumina-Supplied Consumables

- Ligation Buffer (HML)
- 10 mM ATP
- RNA 3' Adapter (RA3)
- RNA 5' Adapter (RA5)
- RNase Inhibitor
- T4 RNA Ligase
- Ultra Pure Water

User-Supplied Consumables

- T4 RNA Ligase 2, Deletion Mutant

Ligate 3' Adapter

[_] 1 Pre-heat the thermal cycler to 70°C and choose the thermal cycler pre-heat lid option and set to 100°C.

[_] 2 Set up the ligation reaction in a sterile, nuclease-free 200 µl PCR tube on ice using the following:

Reagent	Volume (µl)
RNA 3' Adapter (RA3)	1
1 µg Total RNA in Nuclease-free Water	5
**Total Volume**	**6**

[_] 3 Gently pipette the entire volume up and down 6–8 times to mix thoroughly, then centrifuge briefly.

[_] 4 Place the tube on the pre-heated thermal cycler. Close the lid and incubate the tube at 70°C for 2 minutes and then immediately place the tube on ice.

[_] 5 Pre-heat the thermal cycler to 28°C.

[_] 6 Prepare the following mix in a separate, sterile, nuclease-free 200 µl PCR tube on ice. Multiply each reagent volume by the number of samples being prepared. Make 10% extra reagent if you are preparing multiple samples.

Reagent	Volume (µl)
Ligation Buffer (HML)	2
RNase Inhibitor	1
T4 RNA Ligase 2, Deletion Mutant	1
**Total Volume per Sample**	**4**

[_] 7 Gently pipette the entire volume up and down 6–8 times to mix thoroughly, then centrifuge briefly.

[_] 8 Add 4 µl of the mix to the reaction tube from step 2 and gently pipette the entire volume up and down 6–8 times to mix thoroughly. The total volume of the reaction should be 10 µl.

[_] 9 Place the tube on the pre-heated thermal cycler. Close the lid and incubate the tube at 28°C for 1 hour.

[_] 10 With the reaction tube remaining on the thermal cycler, add 1 µl Stop Solution (STP) and gently pipette the entire volume up and down 6–8 times to mix thoroughly. Continue to incubate the reaction tube on the thermal cycler at 28°C for 15 minutes and then place the tube on ice.

Ligate 5' Adapter

[_] 1 Pre-heat the thermal cycler to 70°C.

[_] 2 Aliquot 1.1 x N µl of the RNA 5' Adapter (RA5) into a separate, nuclease-free 200 µl PCR tube, with N equal to the number of samples being processed for the current experiment.

[_] 3 Place the PCR tube on the pre-heated thermal cycler. Close the lid and incubate the tube at at 70°C for 2 minutes and then immediately place the tube on ice.

[_] 4 Pre-heat the thermal cycler to 28°C.

[_] 5 Add 1.1 X N µl of 10mM ATP to the aliquoted RNA 5' Adapter tube, with N equal to the number of samples being processed for the current experiment. Gently pipette the entire volume up and down 6–8 times to mix thoroughly.

[_] 6 Add 1.1 X N µl of T4 RNA Ligase to the aliquoted RNA 5' Adapter tube, with N equal to the number of samples being processed for the current experiment. Gently pipette the entire volume up and down 6–8 times to mix thoroughly.

[_] 7 Add 3 µl of the mix from the aliquoted RNA 5' Adapter tube to the reaction from step 10 of *Ligate 3′ Adapter* on page 3. Gently pipette the entire volume up and down 6–8 times to mix thoroughly.
The total volume of the reaction should now be 14 µl.

[_] 8 Place the tube on the pre-heated thermal cycler. Close the lid and incubate the reaction tube at 28°C for 1 hour and then place the tube on ice.

Reverse Transcribe and Amplify

Reverse transcription followed by PCR is used to create cDNA constructs based on the small RNA ligated with 3' and 5' adapters. This process selectively enriches those fragments that have adapter molecules on both ends. PCR is performed with two primers that anneal to the ends of the adapters.

Illumina-Supplied Consumables

- 25 mM dNTP Mix
- PCR Mix (PML)
- RNA PCR Primer (RP1)
- RNA PCR Primer Index (1–48) (RPI1–RPI48)
  (1 tube of each, depending on the RNA PCR Primer Indices being used)
- RNA RT Primer (RTP)
- RNase Inhibitor
- Ultra Pure Water

User-Supplied Consumables

- 5' and 3' Adapter-ligated RNA (6 µl)
- 5X First Strand Buffer
- 100 mM DTT
- High Sensitivity DNA Chip
- SuperScript II Reverse Transcriptase

Dilute 25 mM dNTP Mix

[_] 1 Pre-heat the thermal cycler to 70°C and choose the thermal cycler pre-heat lid option and set to 100°C.

[_] 2 Dilute the 25 mM dNTPs by premixing the following reagents in a separate, sterile, nuclease-free, 200 µl PCR tube. Multiply each reagent volume by the number of samples being prepared. Make 10% extra reagent if you are preparing multiple samples.

Reagent	Volume (µl)
25 mM dNTP mix	0.5
Ultra Pure Water	0.5
**Total Volume per Sample**	**1.0**

[_] 3 Gently pipette the entire volume up and down 6–8 times to mix thoroughly, then centrifuge briefly.

[_] 4 Label the tube “12.5 mM dNTP Mix” and then place it on ice.

Perform Reverse Transcription

[_] 1 Combine the following in a separate, sterile, nuclease-free, 200 µl PCR tube.

Reagent	Volume (µl)
5' and 3' Adapter-ligated RNA	6
RNA RT Primer (RTP)	1
**Total Volume per Sample**	**37.5**

[_] 2 Gently pipette the entire volume up and down 6–8 times to mix thoroughly, then centrifuge briefly.

[_] 3 Place the tube on the pre-heated thermal cycler. Close the lid and incubate the tube at 70°C for 2 minutes and then immediately place the tube on ice.

[_] 4 Pre-heat the thermal cycler to 50°C.

[_] 5 Prepare the following mix in a separate, sterile, nuclease-free, 200 µl PCR tube placed on ice. Multiply each reagent volume by the number of samples being prepared. Make 10% extra reagent if you are preparing multiple samples.

Reagent	Volume (µl)
5X First Strand Buffer	2
12.5 mM dNTP mix	0.5
100 mM DTT	1
RNase Inhibitor	1
SuperScript II Reverse Transcriptase	1
**Total Volume per Sample**	**5.5**

[_] 6 Gently pipette the entire volume up and down 6–8 times to mix thoroughly, then centrifuge briefly.

[_] 7 Add 5.5 µl of the mix to the reaction tube from step 3. Gently pipette the entire volume up and down 6–8 times to mix thoroughly, then centrifuge briefly.
The total volume should now be 12.5 µl.

[_] 8 Place the tube on the pre-heated thermal cycler. Close the lid and incubate the tube at 50°C for 1 hour and then place the tube on ice.

Perform PCR Amplification

[_] 1 Prepare a separate PCR tube for each index used. Combine the following reagents in a separate, sterile, nuclease-free, 200 µl PCR tube placed on ice. Multiply each reagent volume by the number of samples being prepared. Make 10% extra reagent if you are preparing multiple sample

Reagent	Volume (µl)
Ultra Pure Water	8.5
PCR Mix (PML)	25
RNA PCR Primer (RP1)	2
RNA PCR Primer Index (RPIX)	2
**Total Volume per Sample**	**37.5**

[_] 2 Gently pipette the entire volume up and down 6–8 times to mix thoroughly, then centrifuge briefly, then place the tube on ice.

[_] 3 Add 37.5 µl of PCR master mix to the reaction tube from step 8 of *Perform Reverse Transcription* on page 5.

[_] 4 Gently pipette the entire volume up and down 6–8 times to mix thoroughly, then centrifuge briefly and place the tube on ice.
The total volume should now be 50 µl.

[_] 5 Place the tube on the thermal cycler. Close the lid and amplify the tube on the thermal cycler using the following PCR cycling conditions:

- [_] a Choose the thermal cycler pre-heat lid option and set to 100°C.
- [_] b 98°C for 30 seconds
- [_] c 11 cycles of:
  - 98°C for 10 seconds
  - 60°C for 30 seconds
  - 72°C for 15 seconds
- [_] d 72°C for 10 minutes
- [_] e 4°C hold

[_] 6 Run each sample on a high sensitivity DNA chip according to the manufacturer's instructions.

SAFE STOPPING POINT
If you do not plan to proceed to Purify cDNA Construct immediately, the protocol can be safely stopped here. If you are stopping, store the samples at -15° to -25°C overnight or longer. When proceeding, thaw the samples on ice.

Purify cDNA Construct

This process gel purifies the amplified cDNA construct in preparation for subsequent cluster generation. After gel purification, the cDNA is eluted and can be concentrated by ethanol precipitation if desired. Ethanol precipitation will result in a more concentrated final library, at the cost of some yield. Libraries produced without ethanol precipitation may require special handling during denaturation before loading onto a flow cell.

At this point in the protocol, individual libraries with unique indices may be pooled and gel purified together. Combine equal volumes of the library or molar amounts and then load the samples on the gel according to the instructions below. Do not load more than 30 µl of sample per well.

Illumina-Supplied Consumables

- Custom Ladder
- High Resolution Ladder
- Ultra Pure Water

User-Supplied Consumables

- Gel Breaker Tubes
- 5X Novex TBE Buffer
- 5 µm Filter Tube
- 6% Novex TBE PAGE Gel, 1.0 mm, 10 well (1 per 2 sample run)
- Amplified cDNA Construct (50 µl)
- Razor Blade
- DNA Loading Dye
- High Sensitivity DNA Chip
- Ultra Pure Ethidium Bromide 10mg/ml
- Optional items for ethanol precipitation:
  - 10 mM Tris-HCI, pH 8.5
  - 3 M NaOAc, pH 5.2
  - 70% Ethanol, room temperature
  - 100% Ethanol, -15° to -25°C
  - Glycogen
  - Pellet Paint NF Co-Precipitant

Dilute Pellet Paint NF Co-Precipitant

[Optional, for ethanol precipitation only]

[_] 1 Dilute the Pellet Paint NF Co-Precipitant in a separate, sterile, nuclease-free, 200 µl PCR tube. Multiply each reagent volume by the number of samples being prepared, plus 10% extra reagent. Prepare enough pellet paint for a minimum of 10 samples to avoid pipetting small volumes.

Reagent	Volume (µl)
1X Pellet Paint NF Co-Precipitant	0.2
Ultra Pure Water	1.8
**Total Volume per Sample**	**2.0**

[_] 2 Gently pipette the entire volume up and down to mix thoroughly, then centrifuge briefly.

[_] 3 Label the tube “0.1X Pellet Paint“.

Run Gel Electrophoresis

[_] 1 Determine the volume of 1X TBE Buffer needed. Dilute the 5X TBE Buffer to 1X for use in electrophoresis.

[_] 2 Assemble the gel electrophoresis apparatus per the manufacturer's instructions.

[_] 3 Mix 2 µl of Custom Ladder with 2 µl of DNA Loading Dye.

[_] 4 Mix 1 µl of High Resolution Ladder with 1 µl of DNA Loading Dye.

[_] 5 Mix all of the amplified cDNA construct, (typically 48–50 µl) with 10 µl of DNA Loading Dye.

[_] 6 Load 2 µl of mixed Custom Ladder and loading dye in two wells on the 6% PAGE Gel.

[_] 7 Load 2 µl of High Resolution Ladder and loading dye in a different well.

[_] 8 Load two wells with 25 µl each of mixed Amplified cDNA Construct and loading dye on the 6% PAGE Gel. A total volume of 50 µl should be loaded on the gel.

[_] 9 Run the gel for 60 minutes at 145 V or until the blue front dye exits the gel. Proceed immediately to the next step.

[_] 10 Remove the gel from the apparatus.

Recover Purified Construct

[_] 1 Open the cassette according to the manufacturer's instructions and stain the gel with Ethidium Bromide (0.5 µg/ml in water) in a clean container for 2–3 minutes.

[_] 2 Place the gel breaker tube into a sterile, round-bottom, nuclease-free, 2 ml microcentrifuge tube.

[_] 3 View the gel on a Dark Reader transilluminator or a UV transilluminator.

[_] 4 Using a razor blade, cut out the bands from both lanes that correspond approximately to the adapter-ligated constructs derived from the 22 nt and 30 nt small RNA fragments. Align the razor blade with the top of the 160 bp band of the Custom Ladder, then with the bottom of the 145 bp band of the Custom Ladder. Excise the gel fragment by connecting these cuts on the sides. Both lanes can be combined into one slice.
The band containing the 22 nt RNA fragment with both adapters are a total of 147 nt in length. The band containing the 30 nt RNA fragment with both adapters are 157 nt in length.

[_] 5 Place the band of interest into the 0.5 ml Gel Breaker tube from step 2.

[_] 6 Centrifuge the stacked tubes to 20,000 xg in a microcentrifuge for 2 minutes at room temperature to move the gel through the holes into the 2 ml tube. Ensure that the gel has all moved through the holes into the bottom tube.

[_] 7 If precipitating, proceed to *Concentrate Final Library by Ethanol Precipitation* on page 11. If not precipitating, add 200 µl of Ultra-Pure Water to the gel debris in the 2 ml tube.

[_] 8 Elute the DNA by rotating or shaking the tube at room temperature for at least 2 hours. The tube can be rotated or shaken overnight, if desired.

[_] 9 Transfer the eluate and the gel debris to the top of a 5 µm filter.

[_] 10 Centrifuge the filter for 10 seconds to 600 xg.
During cluster generation, this library may need to be denatured using the protocol in the DNA Template Storage section of the *TruSeq Small RNA Sample Preparation Guide*.

Concentrate Final Library by Ethanol Precipitation

[Optional, for higher concentration]

[_] 1 Add 300 µl of Ultra Pure Water to the gel debris in the 2 ml tube.

[_] 2 Elute the DNA by rotating or shaking the tube at room temperature for at least 2 hours. The tube can be rotated overnight, if desired.

[_] 3 Transfer the eluate and the gel debris to the top of a 5 µm filter.

[_] 4 Centrifuge the filter for 10 seconds to 600 xg.

[_] 5 Add 2 µl of Glycogen, 30 µl of 3M NaOAc, 2 µl of 0.1X Pellet Paint (optional) and 975 µl of pre-chilled -15° to -25°C 100% Ethanol.

[_] 6 Immediately centrifuge to 20,000 xg for 20 minutes on a benchtop microcentrifuge at 4°C.

[_] 7 Remove and discard the supernatant, leaving the pellet intact.

[_] 8 Wash the pellet with 500 µl of room temperature 70% Ethanol.

[_] 9 Centrifuge to 20,000 xg at room temperature for 2 minutes.

[_] 10 Remove and discard the supernatant, leaving the pellet intact.

[_] 11 Dry the pellet by placing the tube, lid open, in a 37°C heat block for 5–10 minutes or until dry.

[_] 12 Resuspend the pellet in 10 µl 10 mM Tris-HCl, pH 8.5.

[_] 13 Proceed to *Validate Library* on page 13.

Validate Library

Illumina recommends performing the following quality control analysis on your sample library.

[_] 1 Load 1 µl of the resuspended construct on an Agilent Technologies 2100 Bioanalyzer using a DNA specific chip such as the DNA-1000 or High Sensitivity DNA chip. Samples prepared without ethanol precipitation should be run on a High Sensitivity DNA chip due to their low concentration.

[_] 2 Check the size, purity, and concentration of the sample.

DNA Template Storage

The storage concentration of the prepared library will depend on whether the library is ethanol precipitated after gel purification. Samples that are not ethanol precipitated can be adjusted to 2 nM and denatured for clustering using the following procedure. Samples that are ethanol precipitated can be adjusted to 10 nM and denatured for clustering using the standard protocol (reference the *cBot User Guide* or *Cluster Station User Guide*).

Adjust the concentration for your prepared DNA samples (or pools of samples) to the desired concentration using Tris-HCI 10 mM, pH 8.5. For long-term storage of DNA samples, add Tween 20 to the sample to a final concentration of 0.1% Tween. This helps to prevent adsorption of the template to plastic tubes upon repeated freeze-thaw cycles, which would decrease the cluster numbers from a sample over time.

Perform the following procedure to denature 2 nM libraries before loading on the flow cell. It uses more volume of the lower-concentration library to denature at the standard 1 nM concentration.

User-Supplied Consumables

- 2 N NaOH
- Hybridization Buffer (HT1) (provided in any Illumina Cluster Generation Kit)
- 10 mM Tris-HCI 10 mM, pH 8.5
- 0.2 ml eight-tube strip

Procedure

[_] 1 Combine the following in a 0.2 ml eight-tube strip:

Reagent	Volume (µl)
2 nM Template DNA	10
Tris-HCI 10 mM, pH 8.5	9
2 N NaOH	1
**Total Volume**	**20**

The template final concentration should be 1 nM.

[_] 2 Vortex briefly to mix the template solution.

[_] 3 Pulse centrifuge the solution.

[_] 4 Incubate for five minutes at room temperature to denature the template into single strands.

[_] 5 Place the denatured DNA template on ice until you are ready to proceed to final dilution.

DNA Template Storage
